# Dysfunction of dimorphic sperm impairs male fertility in the silkworm

**DOI:** 10.1038/s41421-020-00194-6

**Published:** 2020-09-08

**Authors:** Shuqing Chen, Yujia Liu, Xu Yang, Zulian Liu, Xingyu Luo, Jun Xu, Yongping Huang

**Affiliations:** 1 Key Laboratory of Insect Developmental and Evolutionary Biology, Center for Excellence in Molecular Plant Sciences, Shanghai Institute of Plant Physiology and Ecology, Chinese Academy of Sciences, 200032 Shanghai, China; 2 University of Chinese Academy of Sciences, 100049 Beijing, China; 3 Present Address: Department of Genetics, Harvard Medical School, Boston, MA 02115 USA

**Keywords:** Developmental biology, Cell biology

## Abstract

Sperm, which have a vital role in sexual reproduction in the animal kingdom, can display heteromorphism in some species. The regulation of sperm dichotomy remains a longstanding puzzle even though the phenomenon has been widely documented for over a century. Here we use *Bombyx mori* as a model to study a form of sperm dimorphism (eupyrene and apyrene sperm), which is nearly universal among Lepidoptera. We demonstrate that *B. mori Sex-lethal* (*BmSxl*) is crucial for apyrene sperm development, and that *B. mori poly(A)-specific ribonuclease-like domain-containing 1* (*BmPnldc1*) is required for eupyrene sperm development. BmSXL is distributed in the nuclei and cytoplasm of somatic cyst cells in a mesh-like pattern and in the cytoplasm of germ cells enclosed in spermatocysts and sperm bundles. Cytological analyses of dimorphic sperm in *BmSxl* mutants (∆*BmSxl*) showed deficient apyrene sperm with abnormal nuclei, as well as loss of motility associated with malformed mitochondrial derivatives. We define the crucial function of apyrene sperm in the process of fertilization as assisting the migration of eupyrene spermatozoa from bursa copulatrix to spermatheca. By contrast, *BmPnldc1* deficiency (∆*BmPnldc1*) caused eupyrene sperm abnormalities and impaired the release of eupyrene sperm bundles during spermiation. Although apyrene or eupyrene sperm defects impaired fertility of the mutated males, double copulation of a wild-type female with ∆*BmSxl* and ∆*BmPnldc1* males could rescue the sterility phenotypes induced by single copulation with either gene-deficient male. Our findings demonstrate the crucial functions of *BmSxl* and *BmPnldc1* in the development of sperm dimorphism and the indispensable roles of nonfertile apyrene sperm in fertilization.

## Introduction

Remarkable diversity of sperm exist for sexual reproduction in Animalia. Males of some species exhibit sperm polymorphism, regularly producing multiple distinct classes of sperm by a single male^1^. Dichotomous spermatogenesis is found widely in phyla from invertebrates to vertebrates, including Mollusca^1,2^, Annelida^3,4^, Rotifera^5^, Arthropoda^6–8^, Echinodermata^9^, and Chordata^10^. However, the regulatory mechanism for dichotomous spermatogenesis is still unclear at the molecular level. In insecta, sperm dichotomy is a characteristic of lepidopteran species, regularly generating two kinds of co-existing spermatozoa: nucleate (eupyrene) and anucleate (apyrene)^11^. Sperm dichotomy is present throughout Lepidoptera, excluding only two species from the primitive Micropterigidae^12^; however, it is not present in the sister order, Trichoptera^13^, nor in two closely related orders, Diptera and Siphonaptera^14^. Several species of the genus *Drosophila* bear a form of sperm dimorphism termed dimegaly, producing two size classes of nucleated sperm, which is different from the extreme sperm dichotomy seen in Lepidoptera^15–18^.

Like other Lepidoptera, the silkworm, *Bombyx mori*, exhibits dichotomous spermatogenesis. In the testes of normal diploid silkworm males, the developing germ cells are enveloped by the somatic cyst wall cells throughout spermatogenesis^14^. Each cyst contains exclusively eupyrene or apyrene spermatocytes which undergo two successive meiotic divisions to produce 256 spermatids. Following elongation and differentiation, the 256 spermatids develop into eupyrene or apyrene sperm bundles of 256 spermatozoa^11^. Eupyrene sperm bundles, which extrude cytoplasm in a process known as “peristaltic squeezing”, have needle-shaped sperm nuclei located in the anterior part of the elongating cells and contain the typical haploid spermatozoa to fertilize eggs. Apyrene sperm bundles are shorter and completely lack any nuclear material^14^. The round micronuclei located in the middle of the apyrene sperm bundles are squeezed out together with the cytoplasm during the final steps of spermatogenesis.

The nucleated eupyrene sperm are believed to fertilize eggs^8^. The importance of apyrene sperm, which seemed initially to be a kind of “junk” sperm, first gained attention when Sahara and Kawamura demonstrated that double copulation of a normal female silkworm with a highly sterile diploid male (treated with heat-shock to induce abnormal apyrene sperm) and a polyploid male (containing abnormal eupyrene sperm lacking nuclei) recovered fertility^19^. Many hypotheses concerning the function of apyrene sperm have been proposed^18,20^, including possible functions in reducing sperm competition^21^, assisting the eupyrene sperm in spermiation or transport^14,22–24^, facilitating dissociation of eupyrene sperm bundles^25^ and serving as a source of nutrients for the eupyrene sperm, the female, or the zygote^21^. In polyandrous species, apyrene sperm were shown to delay female remating and decrease sperm competition directly or indirectly^26,27^. Still, the mechanism underlying the formation and reproductive function of apyrene sperm remains to be determined.

We previously reported that *Sex-lethal* (*Sxl*), which is a key sex determination factor in *Drosophila melanogaster*, had no effects in somatic sex determination in the lepidopteran insect, *B. mori*, and its physiological function in the silkworm remains further investigation^28^. We also found genes in PIWI-interacting RNA (piRNA) signaling pathway, such as silkworm *Piwi* (*SIWI*) and *B. mori Maelstrom* (*BmMael*), controlled silkworm sexual dimorphic traits, including oogenesis and spermatogenesis^29,30^. We further examined whether genes in these pathways affected the formation of dimorphic sperm. Here our results indicate *B. mori Sex-lethal* (*BmSxl*) and *B. mori poly(A)-specific ribonuclease-like domain-containing 1* (*BmPnldc1*) are crucial for the development of dimorphic sperm. The function of *BmSxl* is very different from that of the *D. melanogaster Sxl* gene, which functions in female individuals as a master-feminizing switch to direct female fate by controlling the dosage compensation system, the somatic sexual differentiation pathway, and germline sex identity^31,32^. Coincidentally, the two homologs show completely different expression patterns, as BmSXL is highly expressed in males, whereas functional *Drosophila* SXL protein is female-specific^31^. PNLDC1 was first identified as Trimmer for piRNA maturation in silkworms^33^, and its mouse homolog is required for meiotic and post-meiotic male germ cell development^34–36^.

Here we used the CRISPR/Cas9 system to generate *BmSxl* mutants (*∆BmSxl*) and *BmPnldc1* mutants (*∆BmPnldc1*). Deficiency of either *BmSxl* or *BmPnldc1* induced sterility of male silkworms, whereas the reproductive system of the female silkworms was unaffected. Loss of *BmSxl* resulted in cytological defects and behavioral abnormalities in apyrene sperm, while the eupyrene sperm were normal. We suggest that defects in mitochondrial derivatives led to complete loss of motility of mutated apyrene spermatozoa in the female genital tract. The normal eupyrene spermatozoa from *∆BmSxl* males were incapable of migrating in the female reproductive tract without the assistance of motile apyrene spermatozoa. So we define the function of active apyrene sperm to assist the migration of eupyrene spermatozoa from the bursa copulatrix to the spermatheca. In contrast to *BmSxl*, we demonstrate that *BmPnldc1* deficiency led to abnormalities in morphology and behavior of eupyrene sperm, whereas the apyrene sperm were normal. Through double copulation of a wild-type female with *∆BmSxl* and *∆BmPnldc1* males, eupyrene sperm from the *∆BmSxl* male and apyrene sperm from the *∆BmPnldc1* male were mixed in the female reproductive tract and significantly recovered the fertility. Altogether, these data reveal that *BmSxl* and *BmPnldc1* have key roles in maintaining normal functions of apyrene sperm and eupyrene sperm. Our results shed light on the function of anucleate nonfertile sperm, and contribute to an understanding of the molecular basis underlying dichotomous spermatogenesis.

## Results

### Expression of *BmSxl* in spermatocysts and sperm bundles

We previously showed that mutation of *BmSxl* had no physiological or morphological effects on somatic sexual determination^28^. To further determine functions of *BmSxl*, we examined the expression of *BmSxl* in different tissues and stages. In the silkworm, there are four *BmSxl* variants: *BmSxl*-*S*^37^, *BmSxl-L*^37^, *BmSxl-1* (accession number: DQ209268) and *BmSxl-2* (accession number: DQ209269). Using the cDNA of testes from fifth instar larvae as template, we performed PCR amplification of the *BmSxl* open reading frames (ORF) and obtained four fragments, 804 base pairs (bp), 873 bp, 942 bp, and 1011 bp in length, which we confirmed by DNA sequencing as four *BmSxl* variants (Supplementary Fig. S1a). We investigated the expression profiles of these four *BmSxl* variants in seven tissues in males and females on day four of the fifth larval instar (L5D4) stage and at the spinning stage using qRT-PCR, and detected high levels of *BmSxl* in the testes at both L5D4 and spinning stages (Supplementary Fig. S1b, c). By dissecting testes, we separated sperm (include spermatocysts and sperm bundles) from testis carcass (empty testis lobuli or follicles), and investigated *BmSxl* expression levels in these two regions from the beginning of the fifth instar (0 h) to the adult stage (360 h). We found that *BmSxl* was highly expressed in sperm, whereas it was expressed at significantly lower levels in the testis carcass at all time points (Supplementary Fig. S1d).

**Fig. 1:**
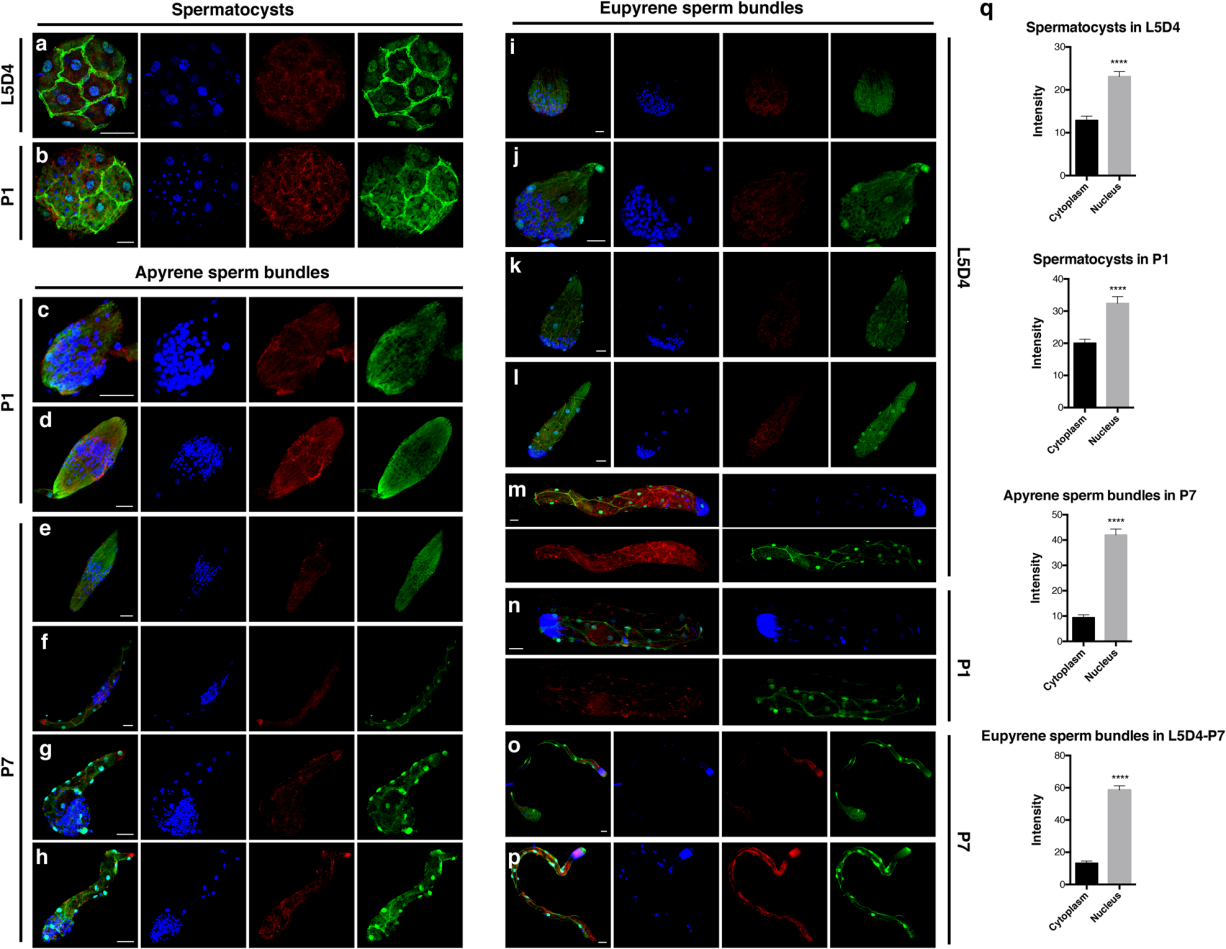
BmSXL is enriched in nuclei and distributed in a mesh-like pattern in the cytoplasm of the somatic cyst cells enveloping the spermatids during spermiogenesis. **a**, **b** Representative immunofluorescence images of spermatocysts at day four of the fifth larval instar (L5D4) stage and pupal stage day 1 (P1). **c**–**f** Representative images of elongating apyrene sperm bundles at stage P1 and P7. **g**, **h** Representative images of apyrene sperm bundles undergoing peristaltic squeezing process at stage P7. **i**–**m** Representative images of elongating eupyrene sperm bundles at stage L5D4. **n** Representative images of a eupyrene sperm bundle with elongating nuclei at stage P1. **o** Representative images of a eupyrene sperm bundle undergoing peristaltic squeezing at stage P7. **p** Representative images of a eupyrene sperm bundle after peristaltic squeezing at stage P7. Blue, Hoechst; red, filamentous actin (F-actin); green, BmSXL. Scale bars, 20 µm (**a**–**p**). **q** Average intensity distribution for BmSXL in the cytoplasm and nucleus of somatic cyst cells in the spermatocysts and sperm bundles. Between 30 and 50 cells (*n* = 30~50) from 10 spermatocysts or sperm bundles were analyzed for each stage. Data are mean ± SEM (****p < 0.0001, paired *t*-test).

The eupyrene and apyrene sperm of silkworm have different morphology, timing of differentiation, and behavior during spermiogenesis^38–40^. Early eupyrene sperm bundles mainly appear in the fifth larval instar, exhibiting well organized round or spearhead-shaped sperm nuclei in the anterior part of the bundles. Late eupyrene sperm bundles are prevalent in the pupal stage, presenting needle-shaped sperm nuclei regularly assembled at the head of bundles. The elongation of apyrene sperm starts later, just before spinning. Elongated apyrene sperm bundles are produced mainly during the pupal stage, maintaining round sperm nuclei in the middle of the bundles until being discarded by peristaltic squeezing^40^. We performed immunofluorescence analyses of spermatocysts and sperm bundles isolated from dissected testes in L5D4, and pupal stages day 1 (P1) and 7 (P7) to gain insight into the intracellular localization of BmSXL in detail. We observed that BmSXL was distributed in a mesh-like pattern in cytoplasm and intensely localized in nuclei of the somatic cyst wall cells (Fig. 1). BmSXL also partially overlapped with filamentous actin (F-actin) in the cell cortex of the somatic cyst cells (Fig. 1a–p; Supplementary Fig. S2). Whereas in germ cells, BmSXL was absent from nuclei but was expressed in the cytoplasm during spermiogenesis (Supplementary Fig. S3). These results demonstrate that BmSXL is located in the spermatocysts and sperm bundles, suggesting that *BmSxl* may function in the dimorphic sperm.

### *BmSxl* is essential for male fertility

**Fig. 2:**
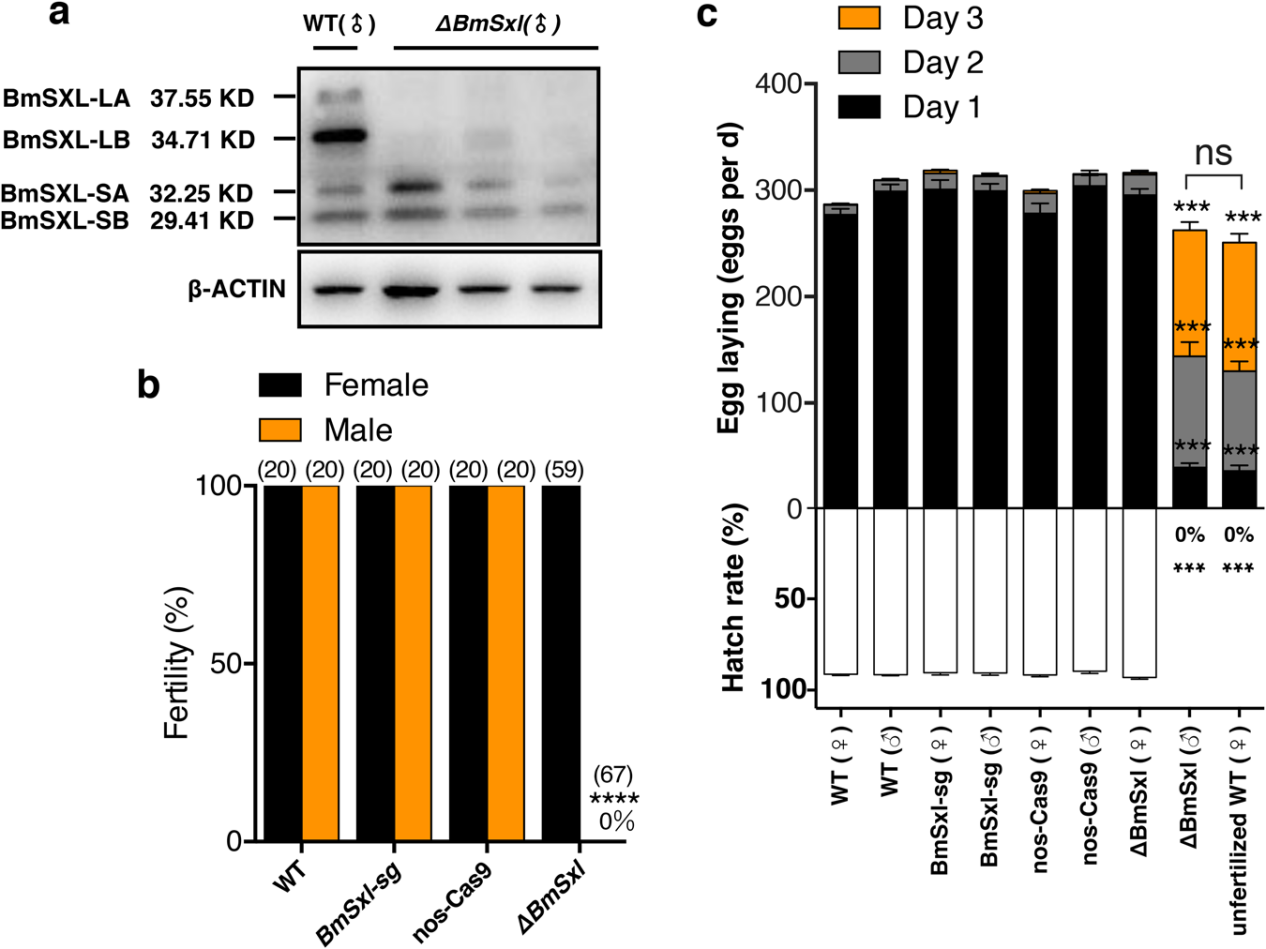
*BmSxl* deficiency leads to male sterility. **a** Western blot analyses of BmSXL protein levels in the testes of adult WT and *∆BmSxl*. β-actin was used as an internal control. **b** Fertility of males and females of indicated genotypes. *∆BmSxl* males were completely sterile (Fertility = 0%). Numbers in parentheses indicate number of pairs tested. ****p < 0.0001, Fisher exact test. **c** Amount of eggs laid and hatch rates of indicated genotypes and genders. Data are mean ± SEM (*n* = 20, ***p < 0.001, Tukey–Kramer HSD statistical test). ns not significant.

To elucidate the role of *BmSxl*, we generated loss-of-function mutants of *BmSxl* using a binary transgenic CRISPR/Cas9 system (Supplementary Fig. S4a, b). We obtained three *BmSxl*-sg12 and two *BmSxl*-sg34 transgenic lines. We generated *∆BmSxl* by crossing the U6-sgRNA (small guide RNA) lines with the nos-Cas9 lines and confirmed that the *BmSxl* gene was successfully disrupted using PCR-based genomic mutation detection (Supplementary Fig. S4c). Use of western blotting confirmed that BmSXL protein levels were significantly decreased in testes of *∆BmSxl* (Fig. 2a). We detected bands in the size range of BmSXL-SA and BmSXL-SB in *∆BmSxl* males, which might be the small-fragment mutated proteins of BmSXL-SA and BmSXL-SB, or truncated proteins of BmSXL-LA and BmSXL-LB (Fig. 2a). The *∆BmSxl* adults were viable and grossly normal. Fecundity tests performed to determine whether the loss of *BmSxl* affected fertility revealed that *∆BmSxl* males were completely sterile (*n* = 67), whereas female mutants retained full fertility (*n* = 59) (Fig. 2b). Wild-type (WT) virgin females mated with WT virgin males laid ~97% of their total laid eggs in the first day after copulation (298.90 ± 6.53 eggs, *n* = 20), followed by gross tapering off in day 2 (10.30 ± 1.68 eggs, *n* = 20) and day 3 (0.50 ± 0.26 eggs, *n* = 20). Of the total eggs laid by WT females mated to WT males, 91.71 ± 0.54% (*n* = 20) hatched after 10 days post-mating (dpm) (Fig. 2c). In contrast, the WT virgin females mated with *∆BmSxl* virgin males laid a few scattered eggs on day 1 (39.45 ± 4.11 eggs, *n* = 20), followed by ~85% of the eggs on day 2 (104.30 ± 13.42 eggs, *n* = 20), and day 3 (118.65 ± 7.79 eggs, *n* = 20) (Fig. 2c). This was similar to the egg-laying behavior of unmated females (day 1, 36.20 ± 5.15 eggs; day 2, 93.70 ± 9.33 eggs; day 3, 120.75 ± 8.49 eggs. *n* = 20) (Fig. 2c). Eggs laid by both WT females mated with *∆BmSxl* males and unmated females failed to hatch after 10 dpm and became shriveled after 20 dpm (Fig. 2c). In contrast, the fertility, egg-laying behavior and hatch rates of *∆BmSxl* females mated to WT males were indistinguishable from those of WT females mated to WT males (Fig. 2b, c). The *∆BmSxl* obtained from *BmSxl*-sg12 × nos-Cas9 and *BmSxl*-sg34 × nos-Cas9 exhibited essentially identical phenotypes; therefore, for the preceding data and hereafter, we used the former as a representative line. These results demonstrate that *BmSxl* is specifically required for the fertility in males.

### *BmSxl* is required for the development of apyrene sperm bundles

**Fig. 3:**
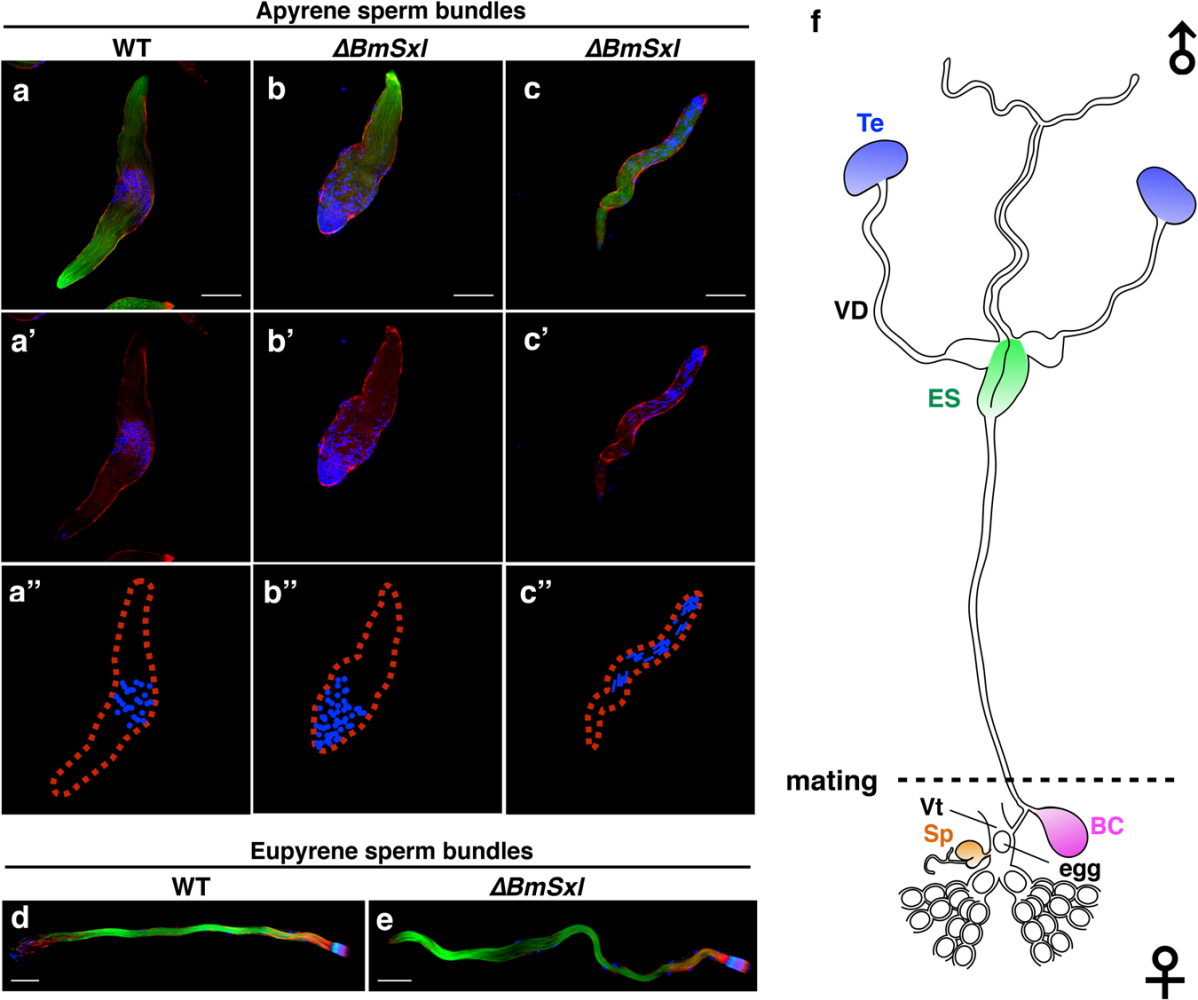
*BmSxl* deficiency impairs the development of apyrene sperm bundles. **a**–**c** Immunostaining images of apyrene sperm bundles in testes of WT and *∆BmSxl* males in P8. **a**–**a″** WT apyrene sperm bundles with round nuclei in the middle section. **b**–**b″** *∆BmSxl* apyrene sperm bundles with round nuclei scattered at one end. **c**–**c″** *∆BmSxl* apyrene sperm bundles with spearhead-shaped, scattered nuclei. **a″**–**c″** Diagrams illustrating the nuclei in sperm bundles. **d**, **e** Immunostaining images of eupyrene sperm bundles in the testes of WT and *∆BmSxl* males in P8. Blue, Hoechst; red, F-actin; green, α-tubulin. Scale bars, 50 µm (**a**–**e**). **f** Diagrams illustrating the male (top) and female (bottom) genital tracts. Te testis, VD vas deferens, ES ejaculatory seminalis, BC bursa copulatrix, Sp spermatheca, Vt vestibulum. Sperm migration path: Te-VD-ES-BC-Sp-Vt-egg entry.

We determined previously that neither the genitalia nor the reproductive system of *∆BmSxl* males exhibited any gross anatomical abnormalities^28^. We also confirmed that *∆BmSxl* males had no difference in several mating behaviors, including mating success, initiation and duration relative to controls (Supplementary Fig. S5a–c). We further examined the cytological phenotype of the sperm bundles in testes of *∆BmSxl* males. During the late pupal stages (from P6 to P8), the eupyrene and apyrene sperm bundles were more easily recognizable, and immunostaining for tubulin, actin and nuclei revealed clear images for the cytological appearance of eupyrene and apyrene sperm bundles. In P8, WT cysts of elongated apyrene spermatids had no polarity, with distribution of small round micronuclei in the middle region (Fig. 3a, a″). By contrast, all the apyrene sperm bundles from *∆BmSxl* males exhibited dramatic defects in sperm nucleus shape and location, and the defective sperm bundles displayed intermediate morphology between WT apyrene sperm bundles and early elongating WT eupyrene sperm bundles (Fig. 3b, c″; Supplementary Fig. S6). Although the lengths of the mutated apyrene sperm bundles were comparable to those of WT apyrene sperm bundles, the sperm nuclei were scattered preferentially in one end of the mutated apyrene sperm bundles in *∆BmSxl* males (Fig. 3b, c″; Supplementary Fig. S6). In addition, the mutated apyrene sperm bundles contained round or spearhead-shaped nuclei which resembled nuclei formed in early elongating WT eupyrene sperm bundles in the fifth larval instar, but without proper location and organization (Supplementary Fig. 6). The late eupyrene sperm bundles were highly polarized with nuclei regularly distributed at one end and sperm tails extending to the other, and showed no cytological differences between *∆BmSxl* and WT in P8 (Fig. 3d, e). These findings indicate that *BmSxl* deficiency specifically leads to aberrant development of apyrene sperm bundles. These results are supported by a recent independent study, also revealing that *BmSxl* is indispensable for the proper morphogenesis of apyrene sperm^41^.

### Apyrene spermatozoa motility is necessary for eupyrene spermatozoa transfer to the spermatheca

During spermiation, the spermatozoa move from the testes toward the male genital duct. At the beginning of spermiation, the apyrene sperm bundles alone are disrupted to liberate individual spermatozoa into the vas deferens. Afterwards, eupyrene and apyrene sperm bundles migrate simultaneously, with the former maintaining the bundled state while the latter disperse. Both types of sperm participate in migration from the male vas deferens to the female spermatheca during copulation. Eupyrene sperm bundles and apyrene spermatozoa are transferred to a single spermatophore within the bursa copulatrix of the female, where the eupyrene sperm bundles are fully dissociated and the apyrene spermatozoa acquire motility. Subsequently, the spermatozoa leave the bursa copulatrix to enter the spermatheca in which apyrene spermatozoa degenerate before oviposition, leaving only eupyrene spermatozoa to be injected into the descending ova where fertilization occurs^42^ (Fig. 3f).

**Fig. 4:**
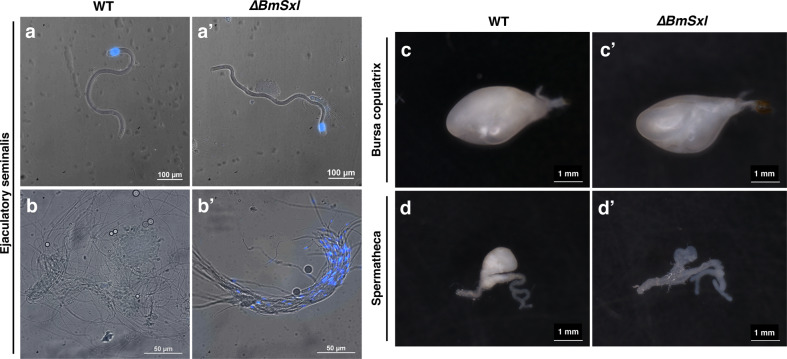
BmSXL lacking results in defective behavior of spermatozoa in adult male and female reproductive tracts. **a**, **a′** Eupyrene sperm bundles in ejaculatory seminalis of unmated WT and *∆BmSxl* males. Blue, Hoechst. **b**, **b′** Apyrene spermatozoa in the ejaculatory seminalis of unmated WT and *∆BmSxl* males. Blue, Hoechst. **c**, **c′** Bursa copulatrix of females mated with WT and *∆BmSxl* males. **d**, **d′** Spermatheca of females mated with WT and *∆BmSxl* males. Scale bars, 100 µm (**a**, **a′**), 50 µm (**b**, **b′**) and 1 mm (**c**–**d′**).

To further characterize the *∆BmSxl* phenotype, we examined the behavior of spermatozoa in male and female reproductive tracts by dissecting the ejaculatory seminalis of unmated WT or *∆BmSxl* males and the bursa copulatrix and spermatheca of WT females mated with virgin WT or *∆BmSxl* males for 4 hours (h) (Fig. 3f). In the ejaculatory seminalis of unmated WT males, the apyrene sperm bundles separated into individual spermatozoa, whereas eupyrene sperm remained in bundles (Fig. 4a, b). In the ejaculatory seminalis of unmated ∆*BmSxl* males, the eupyrene sperm bundles showed no morphological abnormalities (Fig. 4a′), whereas the apyrene sperm bundles were incompletely dissolved with nuclear fragments retained inside the sperm, resembling eupyrene sperm bundles (Fig. 4b′). Both the bursa copulatrix and spermatheca were full in WT females mated with WT males (Fig. 4c, d); nevertheless, although females mated with ∆*BmSxl* males had full bursa copulatrix, their spermatheca were empty (Fig. 4c′, d′). Furthermore, apyrene spermatozoa were shrunken and showed a violent and continuous rotary motion, whereas eupyrene spermatozoa displaying curve shapes were quiescent in the bursa copulatrix of WT females mated with WT males (Supplementary Movie S1). In contrast, shrunken apyrene spermatozoa were rarely observed, and no active apyrene spermatozoa were found in the bursa copulatrix of WT females mated with ∆*BmSxl* males, although motionless eupyrene spermatozoa were abundant (Supplementary Movie S2). These results revealed that *BmSxl* deficiency reduced the efficiency of nuclear eviction and separation of apyrene sperm bundles in the male reproductive system; subsequently, the absence of active apyrene spermatozoa in the female reproductive system caused a failure in migration of spermatozoa from the bursa copulatrix to the spermatheca (BC to Sp, Fig. 3f) and of their subsequent participation in fertilization (Sp-Vt-enter the eggs, Fig. 3f).

### Defective mitochondrial derivatives lead to loss of apyrene spermatozoa motility

**Fig. 5:**
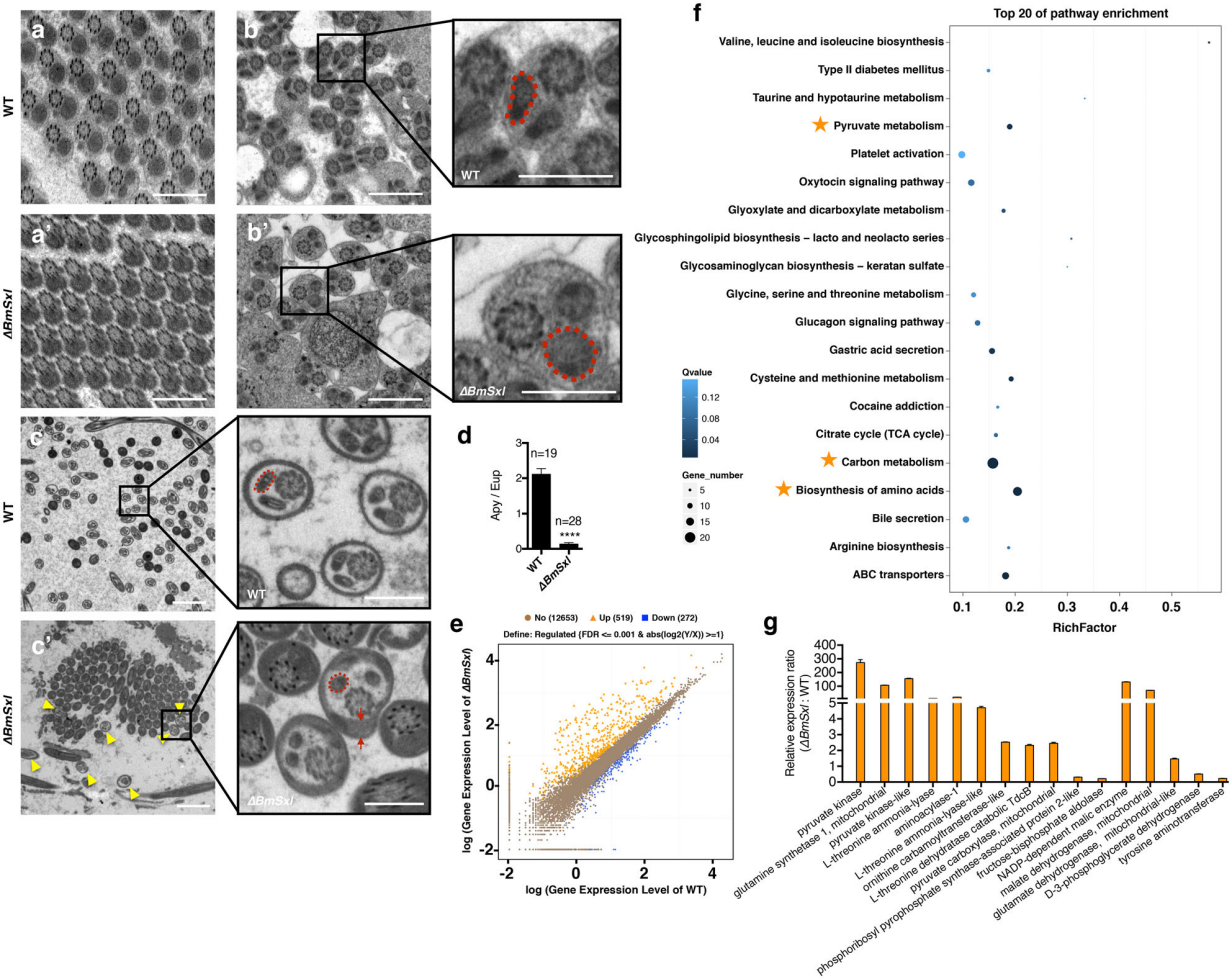
Loss of *BmSxl* leads to defective mitochondrial derivatives in apyrene spermatozoa. **a**, **a′** Transverse sections of eupyrene sperm bundles in testes of WT and *∆BmSxl* adults. **b**, **b′** Transverse sections of apyrene sperm bundles in testes of WT and *∆BmSxl* adults. Red dashed lines mark individual mitochondrial derivatives. Enlargement of **b**, apyrene sperm in WT testes contain mitochondrial derivatives with elliptical transverse sections (marked with red dashed line). Enlargement of **b′**, apyrene sperm in *∆BmSxl* testes contain mitochondrial derivatives with round transverse sections (marked with red dashed line). **c**, **c′** Transverse sections of spermatozoa in the bursa copulatrix of females mated with WT and *∆BmSxl* males. Yellow arrow heads indicated apyrene spermatozoa from *∆BmSxl*. Enlargement of **c**, apyrene spermatozoa from WT contain mitochondrial derivatives with elliptical transverse sections (marked with red dashed line). Enlargement of **c′**, apyrene spermatozoa from *∆BmSxl* with malformed mitochondrial derivatives (marked with red dashed line) and residual electron-dense matrix (pointed by red arrow). Scale bars, 1 µm (**a**–**b′**), 2 µm (**c**, **c′**) and 0.5 µm (in the insets). **d** The ratio of apyrene spermatozoa to eupyrene spermatozoa in the bursa copulatrix of females mated with WT and *∆BmSxl* males. Apy apyrene spermatozoa, Eup eupyrene spermatozoa. Data are mean ± SEM (*n* = sample size, ****p < 0.0001, unpaired *t*-test). **e** Differentially expressed genes (DEGs) in sperm from the testes of WT and *∆BmSxl* adults. DEGs were screened based on the Possion Distribution Method with false discovery rate (FDR) ≤ 0.001 and the absolute value of log2(*Y*/*X*) ≥ 1. **f** The top 20 enriched Kyoto Encyclopedia of Genes and Genomes (KEGG) pathways of DEGs with *p* < 0.05. Orange stars mark three significantly changed metabolic pathways occurring inside mitochondria. **g** Validation of RNA-Seq revealed gene expression changes in the three marked mitochondria metabolic pathways by qRT-PCR. Data are mean ± SEM.

To explore further how *BmSxl* deficiency hinders the transfer of spermatozoa to the spermatheca, we used transmission electron microscopy (TEM) to examine ultrastructural changes of apyrene and eupyrene sperm in the testes and bursa copulatrix. Although the mitochondrial derivatives of the eupyrene sperm bundles were normal, we observed defective mitochondrial derivatives in the apyrene sperm bundles of ∆*BmSxl* male testes, (Fig. 5a–b′). In the testes, the transverse sections of mitochondrial derivatives in WT apyrene sperm bundles were elliptical (Fig. 5b, b′). However, the transverse sections of mitochondrial derivatives in mutated apyrene sperm bundles were much rounder and resembled those in WT eupyrene sperm bundles in the testes (Fig. 5a, b′). The bursa copulatrix of females mated with ∆*BmSxl* males had significantly lower counts of apyrene spermatozoa, and all observed apyrene spermatozoa contained abnormal mitochondrial derivatives (Fig. 5c–d). The transverse sections of mitochondrial derivatives in apyrene spermatozoa from WT males were elliptical and formed “V” configurations, whereas those from ∆*BmSxl* males were round (Fig. 5c, c′). ∆*BmSxl* apyrene spermatozoa also had some electron-dense material that was not present in WT apyrene spermatozoa; in contrast, considerable electron-dense material was contained in WT eupyrene spermatozoa (Fig. 5c, c′). These observations showed that *BmSxl*-deficient apyrene spermatozoa had some intermediate ultrastructural features between normal eupyrene and apyrene spermatozoa.

To gain insights into changes in global gene expression associated with *BmSxl* deficiency, we performed RNA-sequencing (RNA-Seq) on spermatocysts and sperm bundles isolated from testes of WT and ∆*BmSxl* at adult stage (eclosed moths at first day). In a comparison between sperm in testes of ∆*BmSxl* and WT silkworms, we identified 519 significantly upregulated and 272 downregulated genes (Fig. 5e). Consistent with the phenotypic impacts of *BmSxl* deficiency on mitochondrial derivatives, pathway assignments determined with the Kyoto Encyclopedia of Genes and Genomes (KEGG) showed altered gene expression in mitochondrial metabolism pathways, including biosynthesis of amino acids, carbon metabolism and pyruvate metabolism (Fig. 5f). We verified the expression changes of genes involved in mitochondrial metabolism by qRT-PCR (Fig. 5g). These results suggest that the defective ultrastructure of mitochondrial derivatives and the disordered mitochondrial metabolism pathways in ∆*BmSxl* apyrene spermatozoa lead to the loss motility of apyrene spermatozoa and complete male infertility.

### *BmPnldc1* regulates the development of eupyrene sperm

**Fig. 6:**
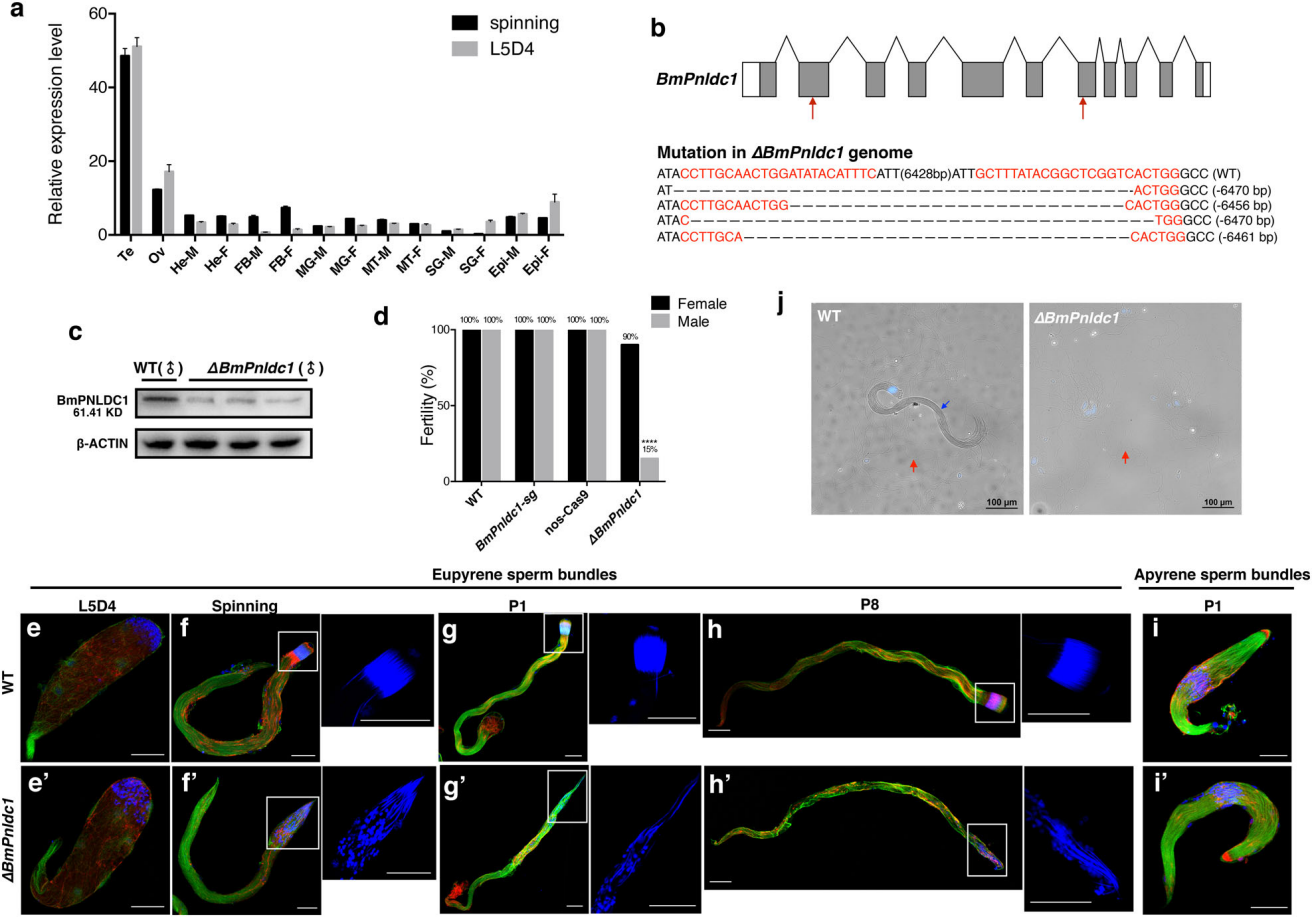
*BmPnldc1* deficiency decreased male fertility due to abnormalities of eupyrene sperm. **a** Expression profile of *BmPnldc1* mRNA at L5D4 and spinning stages. Tissues including testis (Te), ovary (Ov), head (He), fat body (FB), midgut (MG), malpighian tubule (MT), silk gland (SG), epidermis (Epi) from male (-M) and female (-F) were analyzed. Data are mean ± SEM. **b** Genomic disruption of the *BmPnldc1* gene using CRISPR/Cas9. Schematic of *BmPnldc1* gene structure and sgRNA targets. Exons are represented by boxes, ORFs are indicated with gray filled boxes, and 5′ UTRs and 3′ UTRs are shown by blank boxes. Fold lines represent the introns. Introns and other features are not to scale. Red arrows indicate the target sites of sgRNA1 and sgRNA2. Genomic mutations of the *BmPnldc1* gene are shown with target sequences denoted in red. The dashed lines indicate deleted sequences, and indel events are shown to the right. **c** Western blot analyses of BmPNLDC1 protein levels in WT and *∆BmPnldc1* testis in stage L5D4. β-Actin was used as an internal control. **d** Fertility of males and females of indicated genotypes. Fertility is indicated on the histogram. *n* = 20, ****p < 0.0001, Fisher exact test. **e**–**i′** Immunostaining images of sperm bundles from testes of WT and *∆BmPnldc1* males in different developmental stages. Insets show magnification of the anterior part of eupyrene sperm bundles. Blue, Hoechst; red, F-actin; green, α-tubulin. Scale bars, 40 µm. **j** Sperm in ejaculatory seminalis of virgin WT and *∆BmPnldc1* males. A eupyrene sperm bundle is indicated with a blue arrow. Apyrene spermatozoa displaying a shrunken shape are indicated with red arrows. Blue, Hoechst stained nuclei. Scale bars, 100 µm.

piRNAs have a crucial role in germ cells for transposon silencing^43,44^. We found that *BmPnldc1*, a pre-piRNA 3′ Trimmer, is involved in regulating the development of eupyrene sperm. We first investigated the expression profile of *BmPnldc1* in different tissues in L5D4 and spinning stage by qRT-PCR. *BmPnldc1* mRNA was expressed primarily in the testes and ovaries at both the L5D4 and spinning stages (Fig. 6a). We obtained three *BmPnldc1*-sg12 and three *BmPnldc1*-sg34 transgenic lines by silkworm germline transformation. Using the binary transgenic CRISPR/Cas9 system, we generated *∆BmPnldc1* from *BmPnldc1*-sg12 × nos-Cas9 as a representative line. Successful genomic disruptions of *BmPnldc1* were accompanied by a decrease of BmPNLDC1 protein expression in *∆BmPnldc1* testes (Fig. 6b, c). *∆BmPnldc1* animals were viable and grossly normal.

Since *BmPnldc1* is predominantly expressed in gonads, we conducted fecundity tests to evaluate the function of *BmPnldc1*. *∆BmPnldc1* males had significantly lower fertility than WT males, with 85% (17/20) of those tested being sterile (Fig. 6d), while fertility of *∆BmPnldc1* females was not compromised (Fig. 6d). Finding a little fertility among the *∆BmPnldc1* males [15% (3/20)] may have resulted from insufficient disruption of the *BmPnldc1* gene (e.g., non-frameshift mutations) in these individuals. Fluorescent images revealed that the *∆BmPnldc1* sterile males (17/20) all contained defective eupyrene sperm bundles in the testes, whereas the small number of fertile *∆BmPnldc1* males (3/20) had normal eupyrene sperm bundles.

We further investigated spermiogenesis in the testes of L5D4, spinning, P1 and P8 stages in detail (Fig. 6e–i). In the early elongating stage, eupyrene sperm bundles were similar in the *∆BmPnldc1* and WT, with round nuclei in the anterior part of the bundles (Fig. 6e, e′). In the late elongating stages, needle-shaped sperm nuclei were assembled regularly at the head of eupyrene sperm bundles in the WT (Fig. 6f–h). However, at these stages *∆Bmpnldc1* sperm nuclei exhibiting round, spearhead and needle shapes were mingled and scattered in the anterior part of eupyrene sperm bundles (Fig. 6f′–h′), and squeezed eupyrene sperm bundles (SQEs) with abnormally organized sperm nuclei were produced in mutant testes after the peristaltic squeezing process (Fig. 6h′). Moreover, eupyrene sperm bundles were rarely detected in the ejaculatory seminalis of adult *∆BmPnldc1* males, and the rare eupyrene sperm bundles observed all contained malformed sperm nuclei, indicating that the release of eupyrene sperm bundles was largely blocked during spermiation (Fig. 6j). On the other hand, no differences were detected in the apyrene sperm between WT and *∆BmPnldc1* males during spermiogenesis or spermiation (Fig. 6i, j).

We then investigated the behavior of spermatozoa in the female genital tract and found that the bursa copulatrix and spermatheca were plump in females mated with WT males or *∆BmPnldc1* males (Supplementary Fig. S7). Both apyrene and eupyrene spermatozoa were abundant in the bursa copulatrix and spermatheca of females mated with WT males (Supplementary Movies S1 and S3). In contrast, although apyrene spermatozoa were abundant and motile, eupyrene spermatozoa were rare in the bursa copulatrix of females mated with *∆BmPnldc1* males (Supplementary Movie S4). Consistent with this, there were masses of apyrene spermatozoa but few eupyrene spermatozoa in the spermatheca of females mated with *∆BmPnldc1* males (Supplementary Movie S5). Another mutant line, *BmPnldc1*-sg34 × nos-Cas9, exhibited essentially identical phenotypes with *BmPnldc1*-sg12 × nos-Cas9 (Supplementary Fig. S8). Taken together, these results indicate that *BmPnldc1* has an important role in the development of eupyrene sperm, and its loss impairs male fertility.

### Double copulation of a female with *∆BmSxl* and *∆BmPnldc1* males recover fertility

Apyrene sperm was defective in *∆BmSxl*, and eupyrene sperm was dysfunctional in *∆BmPnldc1* males, whereas eupyrene sperm from *∆BmSxl* and apyrene sperm from *∆BmPnldc1* were normal (Figs. 3a–e and 6e–i′). We next asked whether *∆BmSxl* and *∆BmPnldc1* males could recover fertility by double copulation with a WT female. In contrast to finding substantial amounts of eupyrene or apyrene spermatozoa after single copulation with *∆BmSxl* or *∆BmPnldc1* males (Supplementary Movies S2 and S4), we observed masses of eupyrene and apyrene spermatozoa in the bursa copulatrix of females after double copulation with *∆BmSxl* and *∆BmPnldc1* males (Supplementary Movie S6). Instead of the empty spermatheca observed after single copulation with *∆BmSxl* males (Fig. 4d′), the spermatheca were full of both eupyrene and apyrene spermatozoa after additional copulation with *∆BmPnldc1* males (Supplementary Movie S7). These observations indicate that the apyrene spermatozoa contributed by *∆BmPnldc1* males are capable of assisting eupyrene spermatozoa from *∆BmSxl* males to migrate from the bursa copulatrix to the spermatheca in the female reproductive tract.

**Fig. 7:**
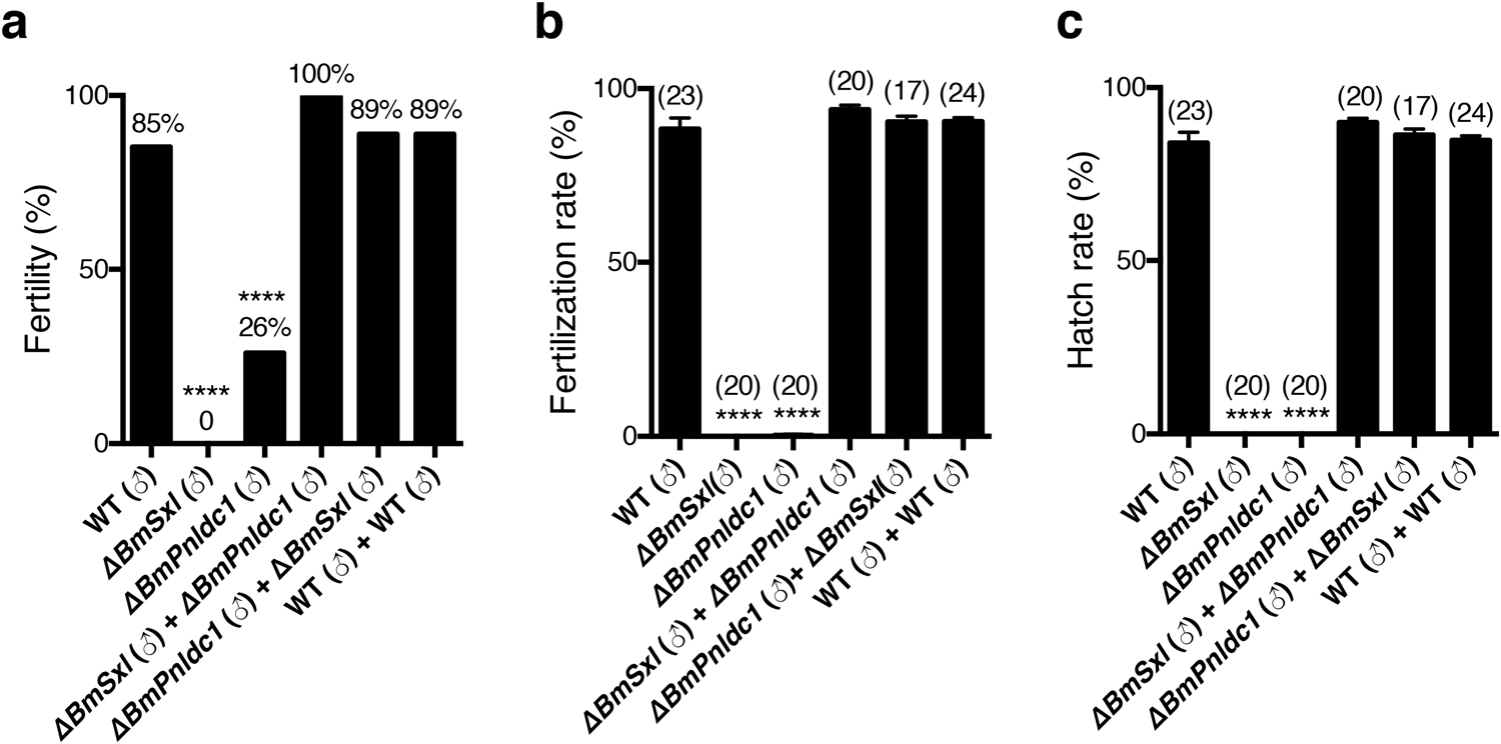
Fertility is recovered by double copulation using *∆BmSxl* and *∆BmPnldc1* males. Analysis of fertility (**a**), fertilization rates (**b**), and hatch rates (**c**) in rescue assays. Fertility indicated on the histogram was scored with all copulation events tested. *n* = 27 for each test, ****p < 0.0001, Fisher exact test. Fertilization rates and hatch rates were analyzed with fertile broods in single or double copulation events of WT males, and with fertility recovered broods in double copulation events and the broods produced in single copulation events by corresponding mutant males; *n* values are shown in parentheses. Data are mean ± SEM, ****p < 0.0001, Tukey–Kramer HSD statistical test. The horizontal axis shows the genotypes of males used in copulation tests. Target females were wild type.

We provided a unique number for every tested individual. In a fertility rescue assay, we conducted a single copulation test by separately mating 27 numbered *∆BmSxl* males (1–27) and 27 numbered *∆BmPnldc1* males (1–27) in sequence with 54 numbered virgin WT females (1–54) for 3 h each (Supplementary Fig. S9a, b). Then in a double copulation test, we mated the *∆BmSxl* males (1–27) with another group of 27 virgin WT females (55–81) for 3 h prior to a second mating of *∆BmPnldc1* males (1–27) with the same females for an additional 3 h (Supplementary Fig. S9c). We also performed a double copulation experiment by mating *∆BmPnldc1* males (1–27) prior to *∆BmSxl* males (1–27) with 82–108 WT virgin females (Supplementary Fig. S9d). Single and double copulation tests were also performed by mating WT males with WT females as controls. Analysis of fertility, fertilization rates and hatch rates for the tests after 10 dpm showed that *∆BmSxl* were completely sterile (100%) and *∆BmPnldc1* were highly sterile (74%) in the single copulation tests (Fig. 7). However, the fertility increased significantly after double copulation of females with both *∆BmSxl* and *∆BmPnldc1* males (Fig. 7a), and the fertilization rates and hatch rates of the recovered broods produced by double copulated females were comparable to controls (Fig. 7b, c). These results indicate that the eupyrene sperm from *∆BmSxl* and the apyrene sperm from *∆BmPnldc1* are functional, and mixing the two in the female reproductive tract by double copulation is sufficient to restore fertility.

## Discussion

In this study, we identify two genes, *BmSxl* and *BmPnldc1*, involved in regulating the development of dimorphic sperm, which are required for male fertility in the silkworm. Loss function of *BmSxl* or *BmPnldc1* induced male infertility due to cytological and behavioral defects of apyrene or eupyrene spermatozoa, respectively. We demonstrate that nonfertile apyrene sperm have an indispensable role in the process of fertilization by assisting the migration of fertile eupyrene sperm in the female reproductive tract. Double copulation of females with ∆*BmSxl* and ∆*BmPnldc1* males significantly rescued the sterile phenotypes induced by single copulation with the mutant males.

The apyrene sperm in ∆*BmSxl* contained abnormal mitochondrial derivatives and showed complete loss of motility, coinciding with the altered expression of genes involved in mitochondrial energy metabolism (Fig. 5; Supplementary Movie S2). We suggest that mitochondrial derivatives in the apyrene sperm host these crucial metabolic pathways, which support the motility of apyrene spermatozoa in the female reproductive tract. Although the eupyrene sperm also contained two mitochondrial derivatives, they could not migrate in the female reproductive tract by themselves. The roles of mitochondrial derivatives of eupyrene sperm in fertilization are still unclear. Most insect sperm bear two elongated mitochondrial derivatives along the flagellum^45–47^. In some insect species, the two mitochondrial derivatives in a single spermatozoon differ in ultrastructural features, such as their size and the material they contain^48,49^. In *Drosophila*, defects in the larger mitochondrial derivatives induce male sterility^50^. In lepidopteran insects, eupyrene and apyrene sperm contain distinctively different mitochondrial derivatives; nevertheless, the molecular constituents and precise functions of the two types of mitochondrial derivatives still remain to be determined^23,40^.

piRNAs act to repress transpositions and protect the integrity of the genome in animal gonads^51–55^. The exonuclease PNLDC1 was first identified in silkworms for shortening the 3′ ends of pre-piRNA^33^. Three recent reports revealed the indispensable role of PNLDC1 in mouse spermatogenesis, and varying frequencies of spermatogenesis arrest were observed in *Pnldc1* mutant mice^34–36^. However, the physiological function of PNLDC1 in silkworms is still unresolved. We demonstrated that loss of function of *BmPnldc1* impaired male fertility due to eupyrene sperm abnormalities (Fig. 6e–j). Although early elongating eupyrene sperm bundles appeared normal in the testes of Δ*BmPnldc1*, they exhibited cytological defects late in spermiogenesis (Fig. 6e–h′). Spermiogenic arrest was not observed in the testis of Δ*BmPnldc1* males, whereas the liberation of eupyrene sperm from the testis to the male reproductive tract was severely blocked during spermiation (Fig. 6j). The eupyrene sperm deficiency had no influence on the morphology or migration behavior of apyrene sperm in Δ*BmPnldc1* males (Fig. 6i, j); although the normal apyrene spermatozoa reached the spermatheca of the females, they were incapable of fertilizing the eggs.

The two kinds of co-occurring spermatozoa, apyrene and eupyrene, differ markedly in their DNA content, morphology, behavior, and differentiation pathways. Apyrene sperm of *∆BmSxl* males had an intermediate morphology between normal apyrene and eupyrene sperm, which was not present in a natural context. The nuclei of *∆BmSxl* apyrene sperm were similar to those of elongated eupyrene sperm in WT males, exhibiting the spearhead-shape and tending to locate at the anterior ends of the sperm bundles. However, the lengths of flagella of the mutated apyrene sperm bundles were unaffected (Supplementary Fig. S6). Like normal eupyrene sperm bundles, the apyrene sperm of *∆BmSxl* males retained nuclei and maintained the bundled state in the ejaculatory seminalis (Fig. 4a, b′). *∆BmSxl* apyrene spermatozoa contained round mitochondrial derivatives similar to normal eupyrene spermatozoa, in addition to a small amount of electron-dense matrix which was absent from normal apyrene spermatozoa but abundant in normal eupyrene spermatozoa (Fig. 5c, c′). These intermediate phenotypes in *∆BmSxl* raise the possibility that *BmSxl* might have a specific role in the switch of eupyrene to apyrene spermatogenesis. Recently, Sakai et al.^41^ also observed abnormal apyrene-like sperm bundles in the testes of *BmSxl* mutants with sperm nuclei similar to those detected during normal eupyrene spermatogenesis. They examined expression patterns of BmSXL protein in cross sections of testicular follicle using a monoclonal antibody against *Drosophila* SXL, and suggested that the expression of *Bm-Sxl-S* in primary spermatocytes may regulate the shift from eupyrene to apyrene spermatogenesis^41^.

We investigated the subcellular distribution of BmSXL in spermatocysts and sperm bundles isolated from testes with antibody against four BmSXL isoforms. BmSXL was located in the somatic-origin cyst cells that encapsulated the germ cells, and partially overlapped with the actin network (Supplementary Fig. S2). Actin is an essential component in the formation of tight junctions, gap junctions and adherent junctions^56–58^. Actin dynamics within somatic cells are reported to facilitate remodeling of cellular junctions around developing germ cells, regulating spermatid maturation and release in the testes of rat, mouse and *Drosophila*^59–63^. In moths, close contact among somatic and germ cells is also facilitated by gap junctions and adherent junctions^64,65^. A possible function of BmSXL is to interact with actin filaments to coordinate the establishment of cellular junctions at the somatic-germ cell interface. In this way the BmSXL-actin network may participate in the decision of bipotential primary spermatocytes to undergo processing into eupyrene or apyrene differentiation pathways by regulating germ-somatic cyst cell communication. Besides, BmSXL contains two highly conserved RNA recognition motifs (RRMs). This suggests another possibility that BmSXL participates in the apyrene–eupyrene shift through binding and exerting post-transcriptional control to its target RNAs. Since BmSXL is distributed in both apyrene and eupyrene sperm bundles, the RNAs or proteins that interacted with BmSXL may be differentially regulated in the two types of sperm bundles to initiate or maintain the sperm dimorphism.

The phenomenon of sperm polymorphism is reported in a wide variety of species in Animalia. Sperm exhibit tremendous diversity in some taxa, and how the dramatic evolutionary divergence occurs remains a mystery. Although discovered over a century ago, the evolutionary rationale for dichotomous spermatogenesis is still unclear. Lepidopteran species, where dichotomous spermatogenesis exists universally, are excellent models for the study of the regulation of sperm dichotomy. Achieving a deeper understanding of dichotomous spermatogenesis in Lepidoptera will enable us to answer these critical but elusive questions.

## Materials and methods

### Silkworm strain

The multivoltine, nondiapausing silkworm strain, Nistari, was used in this study. Larvae were reared on fresh mulberry leaves under standard conditions at 25 °C. Beginning from the fourth molt (set as 0 h), the fifth larval instar stage of the Nistari strain lasted 144 h, followed immediately by the spinning stage. Pupation occurred between 168 and 192 h after the fourth molt. Emergence of adults occurred from 360 h.

### RNA extraction, cDNA synthesis, and quantitative real-time PCR (qRT-PCR)

Total RNA was extracted from silkworm testes and other tissues at different stages using TRIzol reagent (Invitrogen) followed by RNase-free DNAse I (Ambion) treatment according to the manufacturer’s instructions. cDNAs were synthesized using the RevertAid First-Strand cDNA synthesis kit (ThermoFisher Scientific). qRT-PCR analysis was performed on a StepOnePlus Real-Time PCR system (Applied Biosystems) with a SYBR green Real-Time PCR master mix (Toyobo). The *B. mori ribosomal protein 49 gene* (*Bmrp49*) was used as an internal control to standardize the variation of different templates. The amplification program was as follows: samples were incubated at 95 °C for 5 min, followed by 40 cycles of 95 °C for 15 s, and 60 °C for 1 min. Data were analyzed by GraphPad Prism version 6. Sequences of all of the qRT-PCR primers used are listed in Supplementary Table S1. Tests were performed three times.

### Immunofluorescence staining

BmSXL antibodies were generated in rabbits against the full length BmSXL-SB protein and affinity-purified at Youke Biotech. Polyclonal antisera were raised in two rabbits, BmSXL-R1 and BmSXL-R2. The two antisera gave similar western blot signals and immunofluorescence staining patterns. All of the experiments described in this study were performed with antiserum from BmSXL-R2.

Immunofluorescence staining experiments were performed using spermatocysts and sperm bundles isolated from excised testes. The collected sperm were fixed in permeabilizing buffer (1 × PBS + 4% paraformaldehyde + 0.1% Triton X-100 + 0.1% deoxycholate) for 15 min, washed in PBST three times, and subsequently incubated in blocking solution (1 × PBS + 0.1% Triton X-100 + 0.5% bovine serum albumin + 5% normal serum + 1 mM sodium azide) for 30 min. Primary antibody was added to the blocking solution, and then the samples were at 4 °C overnight. After five washes in PBST, samples were incubated with the secondary antibody, Rhodamine-phalloidin and Hoechst for 2 h at room temperature, washed five times with PBST, and subsequently mounted in the antifade medium (YEASEN biotechnology). All images were taken on an Olympus FV1000 microscope.

Quantification of fluorescence intensity of BmSXL in the cytoplasm and nucleus of the somatic cells was performed using ImageJ. The area and integrated optical density in cytoplasm and nucleus of individual somatic cyst cells were measured respectively to obtain mean signal intensity.

Antibodies and their concentrations were as follows: BmSXL-R2, 1:200 (Youke Biotech); mouse-α-tubulin, 1:200 (DM1A, Santa Cruz biotechnology); Alexa Fluor^®^ 488 AffiniPure Goat Anti-Rabbit IgG (H + L), 1:1000 (YEASEN biotechnology); Alexa Fluor^®^ 488 AffiniPure Goat Anti-Mouse IgG (H + L), 1:1000 (YEASEN biotechnology).

### Silkworm germline transformation and CRISPR/Cas9-mediated construction of *BmSxl* and *BmPnldc1*

A binary transgenic CRISPR/Cas9 system was used to construct ∆*BmSxl* and ∆*BmPnldc1*. Construction of the nos-Cas9 transgenic silkworm lines (nos-Cas9/IE1-EGFP) which expressed the Cas9 nuclease under the control of the *B. mori nanos* promoter (nos) was reported previously^28^. The two plasmids for expression of sgRNA targeting *BmSxl* gene under the control of the U6 promoter were constructed to generate *BmSxl*-sg12 (for *BmSxl* target 1 and target 2) and *BmSxl*-sg34 (for *BmSxl* target 3 and target 4) transgenic lines, respectively. Two additional plasmids, which expressed sgRNA targeting the *BmPnldc1* gene were constructed for generating *BmPnldc1*-sg12 (for *BmPnldc1* target 1 and target 2) and *BmPnldc1*-sg34 (for *BmPnldc1* target 3 and target 4) transgenic lines. Primers for plasmid construction and sgRNA targeting sequences are listed in Supplementary Table S1.

The four plasmids were individually injected into preblastoderm G0 embryos with a mixture of helper plasmids and *piggyBac* transposon mRNA. The G0 embryos were subsequently incubated at 25 °C in a humidified chamber for 10–12 days until larval hatching. Putative transgenic G0 moths were mated with WT moths to produce G1 progeny. Screening for transgenic lines carrying the DsRed marker was performed on late G1 embryos using a fluorescence microscope (Nikon, AZ100).

The nos-Cas9 lines and the U6-sgRNA lines were crossed to generate *∆BmSxl* and *∆BmPnldc1* with both EGFP and DsRed fluorescence markers for the following experiments. Genomic DNA extracted from the mutated animals was subjected to PCR amplification with *BmSxl* and *BmPnldc1* specific primers for mutagenesis analysis (Supplementary Table S1). Validation of *BmSxl* knockout efficiency was conducted in the testes of ∆*BmSxl* by western blotting with the BmSXL-R2 antibody (1:1000). Silkworm β-actin, detected by β-actin Rabbit mAb (1:1000; ABclonal), was used as the control. Peroxidase-conjugated Affinipure Goat Anti-Rabbit IgG (H + L) (1:5000; proteintech) was used as the secondary antibody.

### Transmission electron microscopy

Testes and bursa copulatrix were fixed with 2.5% glutaraldehyde in 0.1 M PBS (pH 7.2) overnight at 4 °C. After washing with PBS, the specimens were postfixed with 1% osmium tetroxide in PBS for 1 to 2 h, washed again, and dehydrated with a graded series of ethanol concentrations. The specimens were then embedded in Embed812 resin. Ultrathin sections (50 nm) were stained with 2% uranyl acetate (pH 5.0), followed by 10 mM lead citrate (pH 12), and examined with a Hitachi H-7650 transmission electron microscope.

### RNA-sequencing (RNA-seq) analysis

We tore the testes to release the sperm into the PBS. Washed the sperm in PBS, and then collected the sperm by centrifugation at 5000 rpm for 1 min. Total RNA was extracted from sperm of six adult ∆*BmSxl* and WT males. The mRNA was enriched using Sera-mag Magnetic Oligo (dT) Beads (Illumina), and then fragmented into approximately 200 nt on average, followed by cDNA synthesis with random hexamer primers. After end-repairing the cDNA with phosphate at the 5′ end and stickiness “A” at the 3′ end, ligate the adapters with stickiness “T” at the 3′ end to the cDNA (Illumina). Then 15 cycles of PCR amplification were performed with PCR primers PE 1.0 and PE 2.0, and the libraries were sequenced using the Illumina HiSeq 2000 platform (BGI Genomics). Raw reads obtained from the sequencing were processed by adaptor removal and filtering of low-quality reads, and then mapped to the silkworm genome (http://sgp.dna.affrc.go.jp/KAIKObase/) by tophat/bowtie2. Genes expression level was quantified using the software package called RSEM, and expressed in fragment per kilobase of exon per million fragments mapped (FPKM)^66^. The calculated gene expression levels were then used to compare the difference of gene expression among samples (*Y*/*X*). Differentially expressed genes (DEGs) were screened based on the Possion Distribution Method with false discovery rate (FDR) ≤ 0.001 and the absolute value of log_2_(*Y*/*X*) ≥ 1^67–69^. Enrichment analyses of DEGs were conducted using the Kyoto Encyclopedia of Genes and Genomes (KEGG) database and R packages (clusterProfiler and org.bmor.eg.db).

### Fecundity tests and behavioral assays

Fecundity tests for males were performed by mating single tested virgin males to single WT virgin females for 4 h. Fecundity tests for females were performed by mating single tested virgin females to single WT virgin males for 4 h. Male fertility (%) was measured as the percentage of tested males mated with WT females that gave viable progeny in total tested males^70^. Female fertility (%) was measured as the percentage of tested females mated with WT males that gave viable progeny^70^. The egg fertilization rate (%) was calculated as the percentage of fertilized eggs in total oviposited eggs. The hatch rate (%) was determined as the percentage of eggs that gave viable progeny in the total number of oviposited eggs. The number of viable progeny was determined by counting the number of first instar larvae. After mating the number of eggs laid by each female silkmoth in each test was counted daily over a 3-day period, and the hatch rate was determined 10–12 days later. For detection of mating success, mating initiation and mating duration, each tested male was transferred to a 6-cm-diameter plate containing a WT virgin female. Mating success was calculated as the percentage of tested males that mated with WT virgin females in 5 min. Mating initiation was determined as the time measured from the introduction of a male to a successful mating. Mating duration of more than 4 h (>4 h) was measured as the percentage of tested males that mated with WT females for more than 4 h.

### Single and double copulation in rescue assays

Both males and females were allowed to remate. For a single copulation, a WT virgin female was mated with a tested male for 3 h. For a double copulation test, a WT virgin female was mated with the first male for 3 h, and subsequently mated with the second male for another 3 h. After single or double copulation, females remained in the chambers to lay eggs. The total number of eggs laid by a female in a 3-day period was considered a brood. The fertility, fertilization rate, and hatch rates were determined after 10–12 days post-mating (dpm).

### Statistical analysis

Data were analyzed by GraphPad Prism version 6 and the R software. Error bars represented the mean ± the standard error of the mean (SEM). Statistically significant differences were indicated by asterisks as *p < 0.05, **p < 0.01, ***p < 0.001, and ****p < 0.0001.

**Note**. Please note that as we were preparing the submission of our manuscript, we noticed that Sakai et al.^41^ also found that the functions of *BmSxl* in apyrene sperm formation and eupyrene sperm migration were required for male fertility.

## Supplementary information

Supplementary Information

Supplementary Movie 1

Supplementary Movie 2

Supplementary Movie 3

Supplementary Movie 4

Supplementary Movie 5

Supplementary Movie 6

Supplementary Movie 7

## Supplementary information

**Supplementary Information** accompanies the paper at (10.1038/s41421-020-00194-6).

## References

[1] Hodgson AN (1997). Paraspermatogenesis in gastropod molluscs. Invertebr. Reprod. Dev..

[2] Hodgson AN, Heller J (2000). Spermatozoon structure and spermiogenesis in four species of *Melanopsis* (Gastropoda, Prosobranchia, Cerithioidea) from Israel. Invertebr. Reprod. Dev..

[3] Boi S, Ferraguti M (2001). Temporal pattern of the double sperm line production in *Tubifex tubifex* (Annelida, Oligochaeta). Hydrobiologia.

[4] Ferraguti M, Marotta R, Martin P (2002). The double sperm line in *Isochaetides* (Annelida, Clitellata, Tubificidae). Tissue Cell.

[5] Koehler JK, Birky CW (1966). An electron microscope study of the dimorphic spermatozoa of *Asplanchna* (Rotifera). II. The development of “atypical spermatozoa”. Z. Zellforsch. Mikrosk. Anat..

[6] Meves F (1902). Ueber oligopyrene und apyrene spermien und über ihre Entstehung, nach Beobachtungen an Paludina und Pygaera. Arch. f. Mikrosk. Anat..

[7] Alberti G (2005). Double spermatogenesis in Chelicerata. J. Morphol..

[8] Friedländer M (1997). Control of the eupyrene-apyrene sperm dimorphism in Lepidoptera. J. Insect Physiol..

[9] Eckelbarger KJ, Yong CM, Cameron JL (1989). Ultrastructure and development of dimorphic sperm in the abyssal echinoid *Phrissocystis multispina* (Echinodermata: Echinoidea): implications for deep sea reproductive biology. Biol. Bull..

[10] Hayakawa Y, Komaru A, Munehara H (2002). Ultrastructural observations of eu- and paraspermiogenesis in the cottid fish *Hemilepidotus gilberti* (Teleostei: Scorpaeniformes: Cottidae). J. Morphol..

[11] Phillips DM (1971). Morphogenesis of the lacinate appendages of Lepidopteran spermatozoa. J. Ultrastruct. Res..

[12] Sonnenschein M, Häuser CL (1990). Presence of only eupyrene spermatozoa in adult males of the genus *Micropterix* hübner and its phylogenetic significance (Lepidoptera: Zeugloptera, Micropterigidae). Int. J. Insect Morphol. Embryol..

[13] Friedländer M (1983). Phylogenetic branching of Trichoptera and Lepidoptera: an ultrastructural analysis on comparative spermatology. J. Ultrastruct. Res..

[14] Friedländer M, Seth RK, Reynolds SE (2005). Eupyrene and apyrene sperm: dichotomous spermatogenesis in Lepidoptera. Adv. Insect Phys..

[15] Chang H, Miller DD (1981). Further observations on polymegaly in species of the *Drosophila affinis* subgroup. Trans. Neb. Acad. Sci..

[16] Pasini ME, Redi CA, Caviglia O, Perotti ME (1996). Ultrastructural and cytochemical analysis of sperm dimorphism in *Drosophila subobscura*. Tissue Cell.

[17] Snook RR, Karr TL (1998). Only long sperm are fertilization-competent in six sperm-heteromorphic *Drosophila* species. Curr. Biol..

[18] Swallow JG, Wilkinson GS (2002). The long and short of sperm polymorphism in insects. Biol. Rev. Camb. Philos. Soc..

[19] Sahara K, Kawamura N (2002). Double copulation of a female with sterile diploid and polyploid males recovers fertility in *Bombyx mori*. Zygote.

[20] Osanai M, Kasuga H, Aigaki T (1987). Physiological role of apyrene spermatozoa of *Bombyx mori*. Experientia.

[21] Silberglied RE, Shepherd JG, Dickinson JL (1984). Eunuchs: the role of apyrene sperm in Lepidoptera. Am. Nat..

[22] Holt GG, North DT (1970). Effects of gamma irradiation on the mechanisms of sperm transfer in *Trichoplusia ni*. J. Insect Physiol..

[23] Friedländer M, Gitay H (1972). The fate of the normal-anucleated spermatozoa in inseminated females of the silkworm *Bombyx mori*. J. Morphol..

[24] Katsuno S (1977). Studies on eupyrene and apyrene spermatozoa in the silkworm, *Bombyx mori* L. (Lepidoptera: Bombycidae). I. The intratesticular behaviour of the spermatozoa at various stages from the 5th-instar to the adult. Appl. Entomol. Zool..

[25] Katsuno S (1977). Studies on eupyrene and apyrene spermatozoa in the silkworm, *Bombyx mori* L. (Lepidoptera: Bombycidae). V. The factor related to the separation of eupyrene sperm bundles. Appl. Entomol. Zool..

[26] Cook PA, Wedell N (1999). Non-fertile sperm delay female remating. Nature.

[27] Mongue AJ, Hansen ME, Gu L, Sorenson CE, Walters JR (2019). Nonfertilizing sperm in Lepidoptera show little evidence for recurrent positive selection. Mol. Ecol..

[28] Xu J (2017). *Bombyx mori* P-element somatic inhibitor (BmPSI) is a key auxiliary factor for silkworm male sex determination. PLoS Genet..

[29] Li Z (2018). *Bombyx mori* histone methyltransferase *BmAsh2* is essential for silkworm piRNA-mediated sex determination. PLoS Genet..

[30] Chen K (2019). *Maelstrom* regulates spermatogenesis of the silkworm, *Bombyx mori*. Insect Biochem. Mol. Biol..

[31] Penalva LO, Sánchez L (2003). RNA binding protein Sex-Lethal (Sxl) and control of *Drosophila* sex determination and dosage compensation. Microbiol. Mol. Biol. Rev..

[32] Salz HK, Erickson JW (2010). Sex determination in *Drosophila*: the view from the top. Fly.

[33] Izumi N (2016). Identification and functional analysis of the Pre-piRNA 3’ trimmer in silkworms. Cell.

[34] Ding D (2017). PNLDC1 is essential for piRNA 3’ end trimming and transposon silencing during spermatogenesis in mice. Nat. Commun..

[35] Zhang Y (2017). An essential role for PNLDC1 in piRNA 3’ end trimming and male fertility in mice. Cell Res..

[36] Nishimura T (2018). PNLDC1, mouse pre-piRNA Trimmer, is required for meiotic and post-meiotic male germ cell development. EMBO Rep..

[37] Niimi T (2006). Molecular cloning and chromosomal localization of the *Bombyx Sex-lethal* gene. Genome.

[38] Yamashiki N, Kawamura N (1997). Behaviors of nucleus, basal bodies and microtubules during eupyrene and apyrene spermiogenesis in the silkworm, *Bombyx mori* (Lepidoptera). Dev. Growth Differ..

[39] Yamashiki N, Kawamura N (1998). Behavior of centrioles during meiosis in the male silkworm, *Bombyx mori* (Lepidoptera). Dev. Growth Diff..

[40] Kawamura N, Yamashiki N, Bando H (1998). Behavior of mitochondria during eupyrene and apyrene spermatogenesis in the silkworm, *Bombyx mori* (Lepidoptera), investigated by fluorescence in situ hybridization and electron microscopy. Protoplasma.

[41] Sakai H (2019). Dimorphic sperm formation by *Sex-lethal*. Proc. Natl Acad. Sci. USA.

[42] Katsuno S (1977). Studies on eupyrene and apyrene spermatozoa in the silkworm, *Bombyx mori* L. (Lepidoptera: Bombycidae). IV. The behaviour of the spermatozoa in the internal reproductive organs of female adults. Appl. Entomol. Zool..

[43] Saxe JP, Chen M, Zhao H, Lin H (2013). Tdrkh is essential for spermatogenesis and participates in primary piRNA biogenesis in the germline. EMBO J..

[44] Nishida KM (2018). Hierarchical roles of mitochondrial Papi and Zucchini in *Bombyx* germline piRNA biogenesis. Nature.

[45] Moreira J, Araújo VA, Báo SN, Lino-Neto J (2010). Structural and ultrastructural characteristics of male reproductive tract and spermatozoa in two *Cryptinae* species (Hymenoptera: Ichneumonidae). Micron.

[46] Rego LNAA, Alevi KC, Azeredo-Oliveira MTV, Madi-Ravazzi ,L (2016). Ultrastructural features of spermatozoa and their phylogenetic application in Zaprionus (Diptera, Drosophilidae). Fly.

[47] Werner M, Simmons LW (2008). Insect sperm motility. Biol. Rev. Camb. Philos. Soc..

[48] Rego LNAA, Silistino-Souza R, Azeredo-Oliveira MTVD, Madi-Ravazzi L (2013). Spermatogenesis of *Zaprionus indianus* and *Zaprionus sepsoides* (Diptera, Drosophilidae): cytochemical, structural and ultrastructural characterization. Genet. Mol. Biol..

[49] Gracielle IM, Tidon R, Báo SN (2016). Structure and ultrastructure of spermatozoon in six species of Drosophilidae (Diptera). Tissue Cell.

[50] Laurinyecz B (2019). Sperm-Leucylaminopeptidases are required for male fertility as structural components of mitochondrial paracrystalline material in *Drosophila melanogaster* sperm. PLoS Genet..

[51] Aravin AA, Hannon GJ, Brennecke J (2007). The Piwi-piRNA pathway provides an adaptive defense in the transposon arms race. Science.

[52] Houwing S (2007). A role for Piwi and piRNAs in germ cell maintenance and transposon silencing in Zebrafish. Cell.

[53] Klattenhoff C, Theurkauf W (2008). Biogenesis and germline functions of piRNAs. Development.

[54] Quénerch’du E, Anand A, Kai T (2016). The piRNA pathway is developmentally regulated during spermatogenesis in *Drosophila*. RNA.

[55] Gou LT (2017). Ubiquitination-deficient mutations in human *Piwi* cause male infertility by impairing histone-to-protamine exchange during spermiogenesis. Cell.

[56] Gumbiner BM (2000). Regulation of cadherin adhesive activity. J. Cell Biol..

[57] Halbleib JM, Nelson WJ (2006). Cadherins in development: cell adhesion, sorting, and tissue morphogenesis. Genes Dev..

[58] Qian X (2014). Actin binding proteins, spermatid transport and spermiation. Semin. Cell Dev. Biol..

[59] Lie PP, Chan AY, Mruk DD, Lee WM, Cheng CY (2010). Restricted Arp3 expression in the testis prevents blood-testis barrier disruption during junction restructuring at spermatogenesis. Proc. Natl Acad. Sci. USA.

[60] Xiao X (2014). N-wasp is required for structural integrity of the blood-testis barrier. PLoS Genet..

[61] Rotkopf. S (2011). The WASp-based actin polymerization machinery is required in somatic support cells for spermatid maturation and release. Development.

[62] Dubey P, Shirolikar S, Ray K (2016). Localized, reactive F-actin dynamics prevents abnormal somatic cell penetration by mature spermatids. Dev. Cell.

[63] Dubey P, Kapoor T, Gupta S, Shirolikar S, Ray K (2019). Atypical septate junctions maintain the somatic enclosure around maturing spermatids and prevent premature sperm release in *Drosophila* testis. Biol. Open.

[64] Szöllösi, A. in *Insect Ultrastructure* (eds King, R. C. & Akai, H.) Vol. 1, 32–60 (Springer, Boston, MA, 1982).

[65] Sahara K, Kawamura N (2004). Roles of actin networks in peristaltic squeezing of sperm bundles in *Bombyx mori*. J. Morphol..

[66] Li B, Dewey CN (2011). RSEM: accurate transcript quantification from RNA-Seq data with or without a reference genome. BMC Bioinformatics.

[67] Audic S, Claverie JM (1997). The significance of digital gene expression profiles. Genome Res..

[68] Abdi, H. in *Encyclopedia of Measurement and Statistics* (ed. Salkind, N. J.) 103–107 (Sage, Thousand Oaks, CA, 2007).

[69] Benjamini Y, Yekutieli D (2001). The control of the false discovery rate in multiple testing under dependency. Ann. Stat..

[70] Pavlou HJ (2016). Neural circuitry coordinating male copulation. Elife.

