# Supplementary material for: Dysfunction of dimorphic sperm impairs male fertility in the silkworm
Source: Cell Discov. 2020 Sep 8;6:60. doi: 10.1038/s41421-020-00194-6 (PMC7477584; doi:10.1038/s41421-020-00194-6)
Supplement: Supplementary file 1 — Supplementary Information [file 41421_2020_194_MOESM1_ESM.docx]

**Supplementary Movie S1.** Behavior of spermatozoa in the bursa copulatrix of females mated with WT males. Scale bar, 100 µm.

**Supplementary Movie S2.** Behavior of spermatozoa in the bursa copulatrix of females mated with *∆BmSxl* males. Scale bar, 100 µm.

**Supplementary Movie S3.** Behavior of spermatozoa in the spermatheca of females mated with WT males. Scale bar, 100 µm.

**Supplementary Movie S4.** Behavior of spermatozoa in the bursa copulatrix of females mated with *∆BmPnldc1* males. Scale bar, 100 µm.

**Supplementary Movie S5.** Behavior of spermatozoa in the spermatheca of females mated with *∆BmPnldc1* males. Scale bar, 100 µm.

**Supplementary Movie S6.** Behavior of spermatozoa in the bursa copulatrix of females doubly copulated with *∆BmSxl* males and *∆BmPnldc1* males. Scale bar, 100 µm.

**Supplementary Movie S7.** Behavior of spermatozoa in the spermatheca of females doubly copulated with *∆BmSxl* males and *∆BmPnldc1* males. Scale bar, 100 µm.

**Supplementary Figures**

**
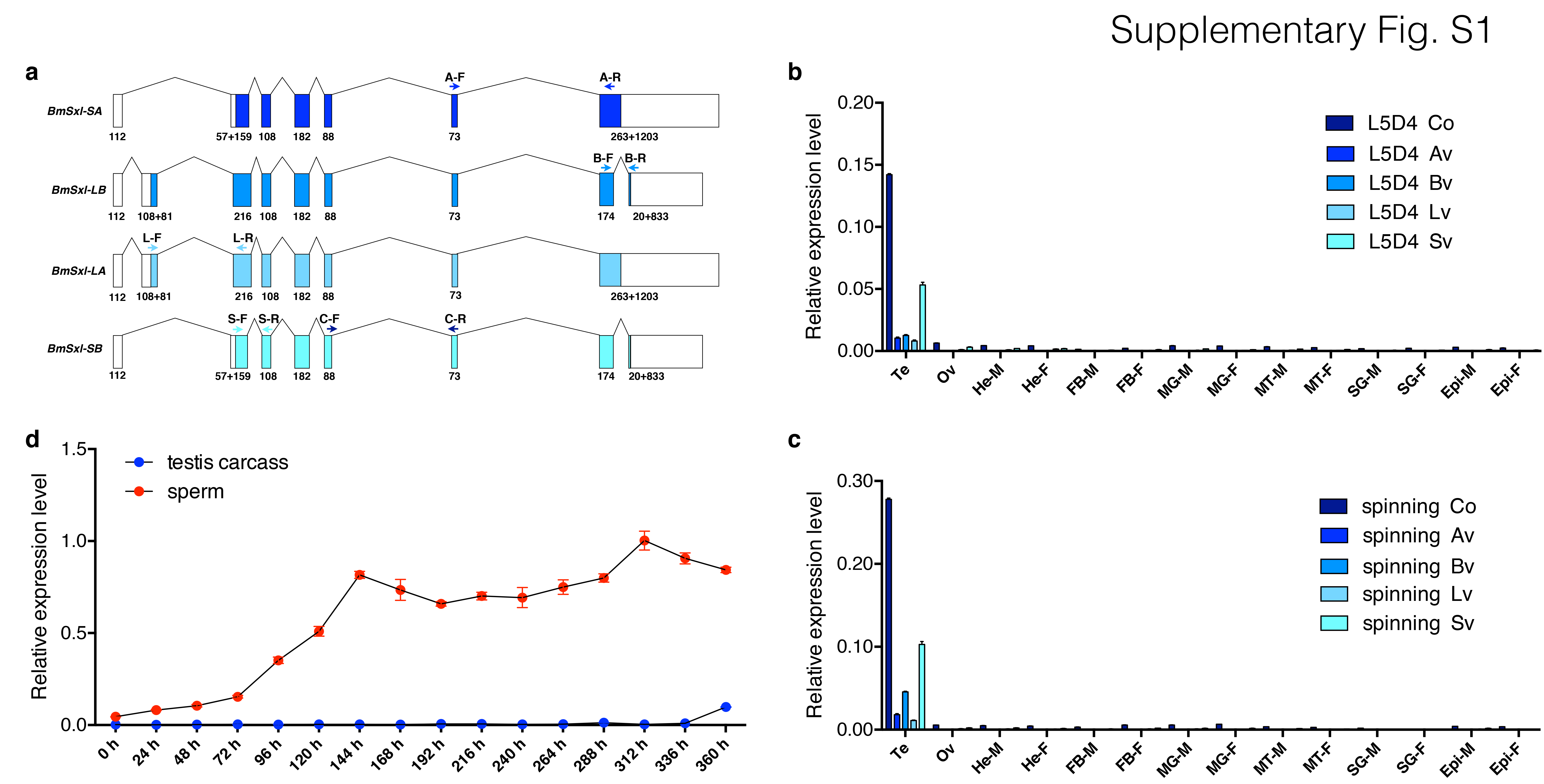
**

**Supplementary Fig. 1 *BmSxl* is predominantly expressed in sperm during spermiogenesis (related to Fig. 1).** **a** The gene structure of *BmSxl*. Boxes represent exons; ORFs are marked with solid filled boxes; 5’ untranslated regions (5’ UTR) and 3’ untranslated regions (3’ UTR) are marked with blank boxes. The sizes of exons are indicated below the boxes. Fold lines represent the intron sequences. Primers used for analysis of the *BmSxl* expression level in (**b**-**d**) are indicated above the boxes. Introns and other features are not to scale. **b**, **c** The relative expression levels of *BmSxl* mRNA at L5D4 and spinning stages. Tissues including testis (Te), ovary (Ov), head (He), fat body (FB), midgut (MG), malpighian tubule (MT), silk gland (SG), epidermis (Epi) from male (-M) and female (-F) were analyzed. Co, Av, Bv, Lv and Sv represent the relative *BmSxl* mRNA expression level determined by qRT-PCR with primers C-F & C-R, A-F & A-R, B-F & B-R, L-F & L-R and S-F & S-R. Data are mean ± SEM. **d** The relative expression level of *BmSxl* mRNA in sperm and testis carcass from 0 h to 360 h after the fourth molt. Testis carcass refers to the whole testis with sperm removed. qRT-PCR was conducted with primers C-F & C-R. Data are mean ± SEM.


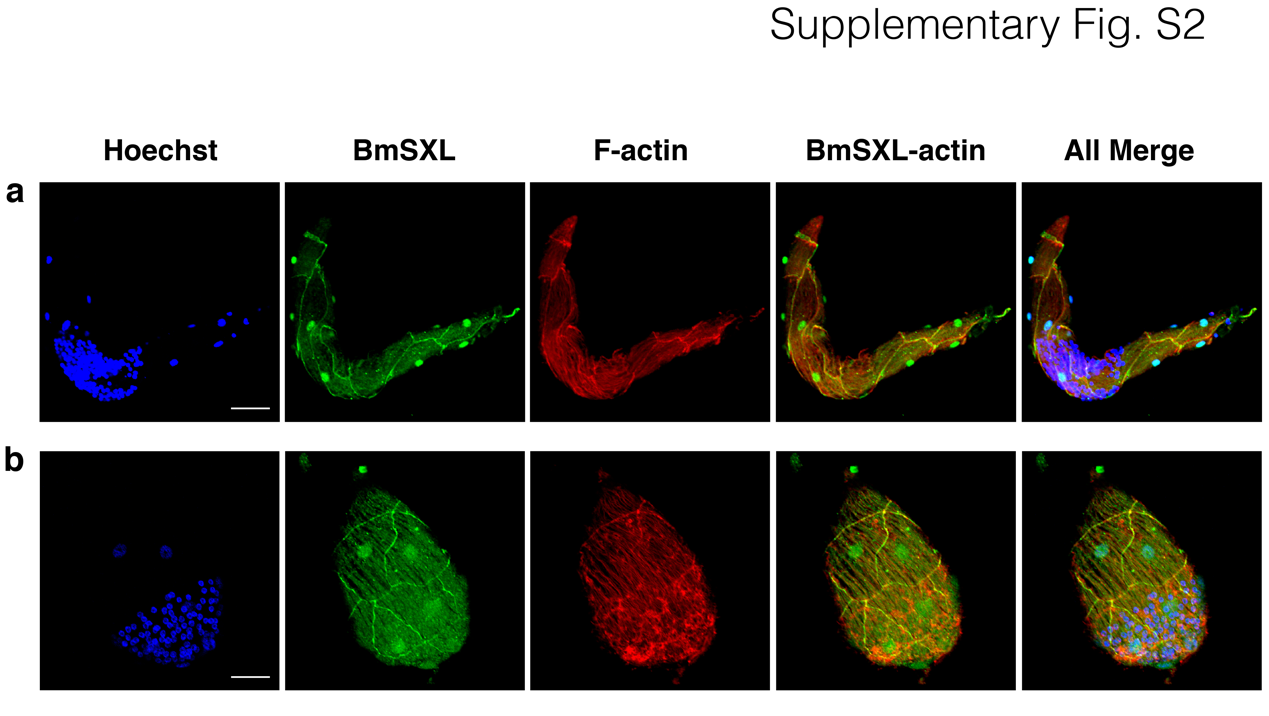


**Supplementary Fig. 2 Partial co-localization of BmSXL and F-actin network in the cell cortex of the somatic cyst cells (related to Fig. 1). a** An apyrene sperm bundle in P1. **b** A eupyrene sperm bundle in L5D4. Blue, Hoechst; red, F-actin; green, BmSXL. Scale bars, 30 µm.


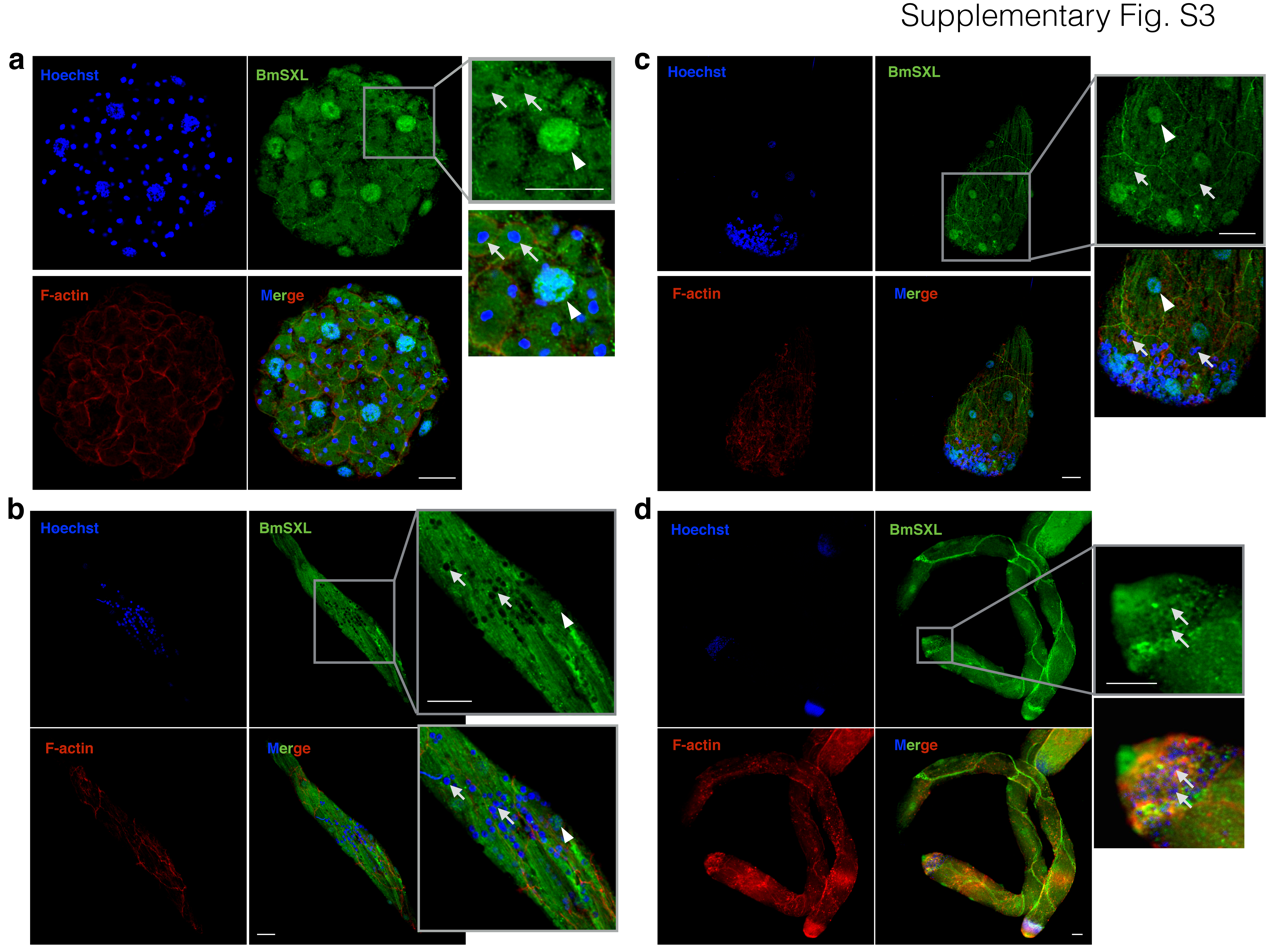


**Supplementary Fig. 3 BmSXL is absent from the nuclei of germ cells (related to Fig. 1). a** A round spermatocyst containing spermatocytes in P1. **b** An elongating apyrene sperm bundle containing elongating apyrene spermatids in P7. **c**, **d** Elongating eupyrene sperm bundles containing elongating eupyrene spermatids in L5D4 and spinning stages. BmSXL is abundant in nuclei of somatic cyst cells (marked with white arrowheads), but absent from nuclei of spermatocytes and spermatids (marked with white arrows). In eupyrene sperm bundles, the absence of BmSXL from spermatid nuclei was not so obvious. Blue, Hoechst; red, F-actin; green, BmSXL. Scale bars, 20 µm **(a**-**d**).


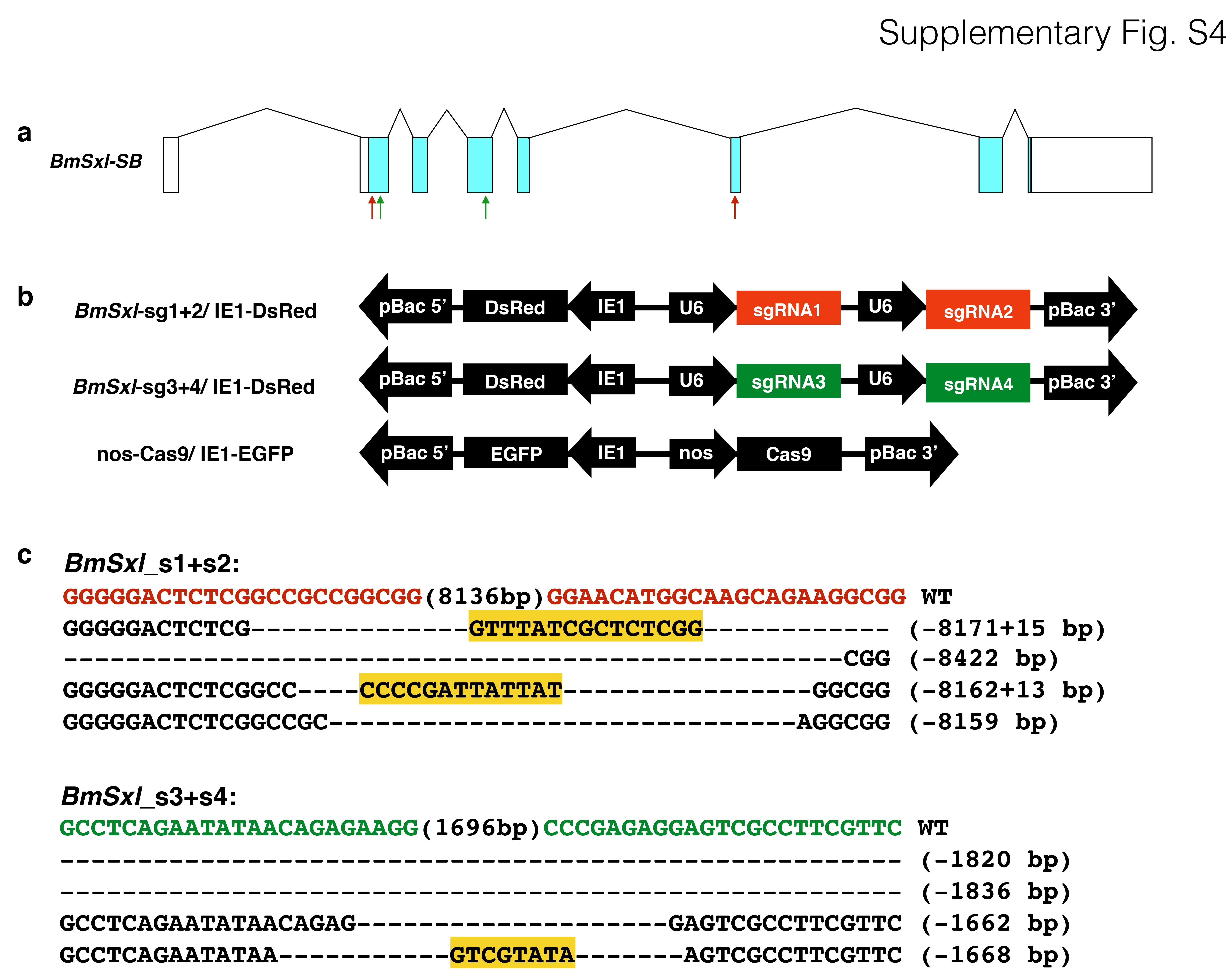


**Supplementary Fig. 4 Construction of *∆BmSxl* by a binary CRISPR/Cas9 system (related to Fig. 2). a** Schematic of the location of sgRNA targeting sequences. Red arrows indicate the target sites of sgRNA1 and sgRNA2. Green arrows indicate the target sites of sgRNA3 and sgRNA4. **b** Design of the plasmids for sgRNA and Cas9 expression. sgRNA expression was driven by the U6 promoter. Cas9 expression was driven by the *nos* promoter. Expression of genes encoding DsRed and EGFP fluorescence markers was driven by the baculovirus *immediate-early 1* gene promoter (IE1). **c** Confirmation of the disruption of *BmSxl* gene using Sanger sequencing. Deletions are shown as dashes, and inserted sequences are highlighted in yellow shadows. Indel mutations are enumerated in parentheses to the right of each sequence (-, insertion; +, deletion). The value in parentheses between the two sgRNA targets refers to the length of the interspace fragment in base pairs.


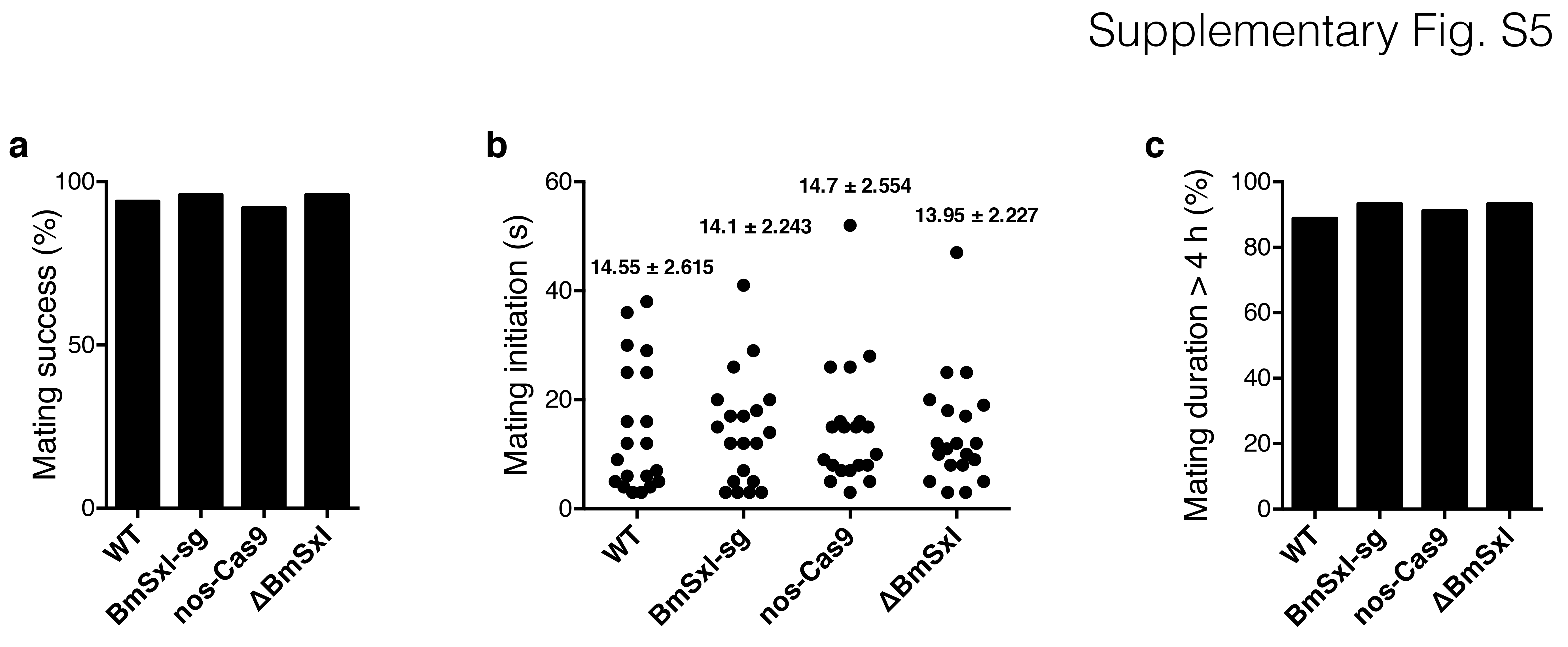


**Supplementary Fig. 5 Male mating behavior (related to Fig. 2). a** Mating success (n = 50, p ≥ 0.05, Fisher exact test). **b** Mating initiation (n = 20, mean ± SEM, p ≥ 0.05, Tukey-Kramer HSD test). **c** Mating duration (n = 45, p ≥ 0.05, Fisher exact test).


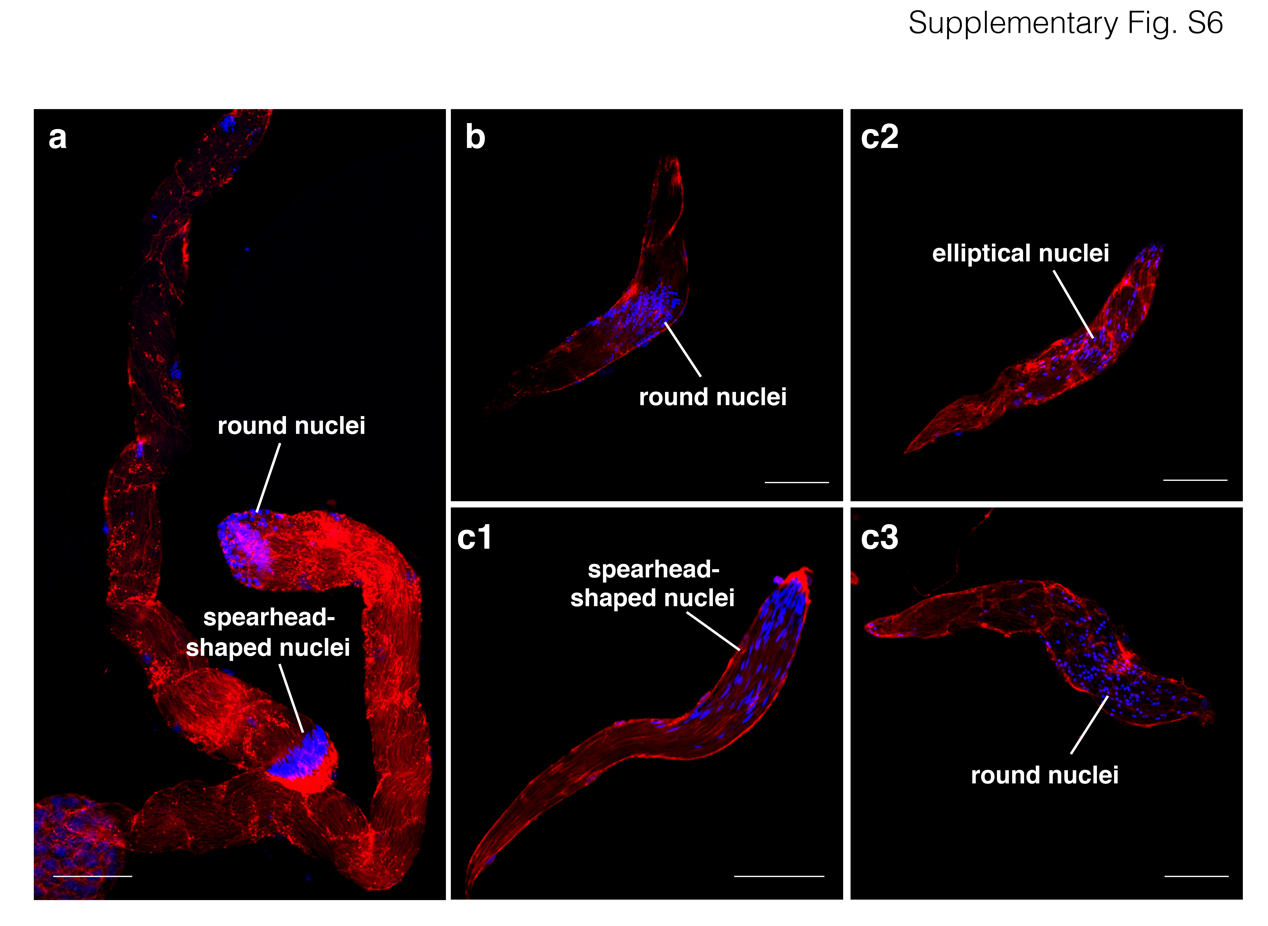


**Supplementary Fig. 6 Apyrene sperm bundles of *∆BmSxl* males show intermediate morphology between normal apyrene and normal eupyrene sperm bundles (related to Fig. 3). a** Early WT eupyrene sperm bundles with round nuclei and spearhead-shaped nuclei in the anterior regions in stage L5D4. **b** WT apyrene sperm bundles with round nuclei located in the middle region in stage P8. **c1**-**c3** *∆BmSxl* apyrene sperm bundles with spearhead-shaped (**c1**), elliptical (**c2**) and round (**c3**) nuclei scattered preferentially in one end of the bundles in stage P8. Blue, Hoechst; red, F-actin. Scale bars, 50 µm (**a**-**c3**).


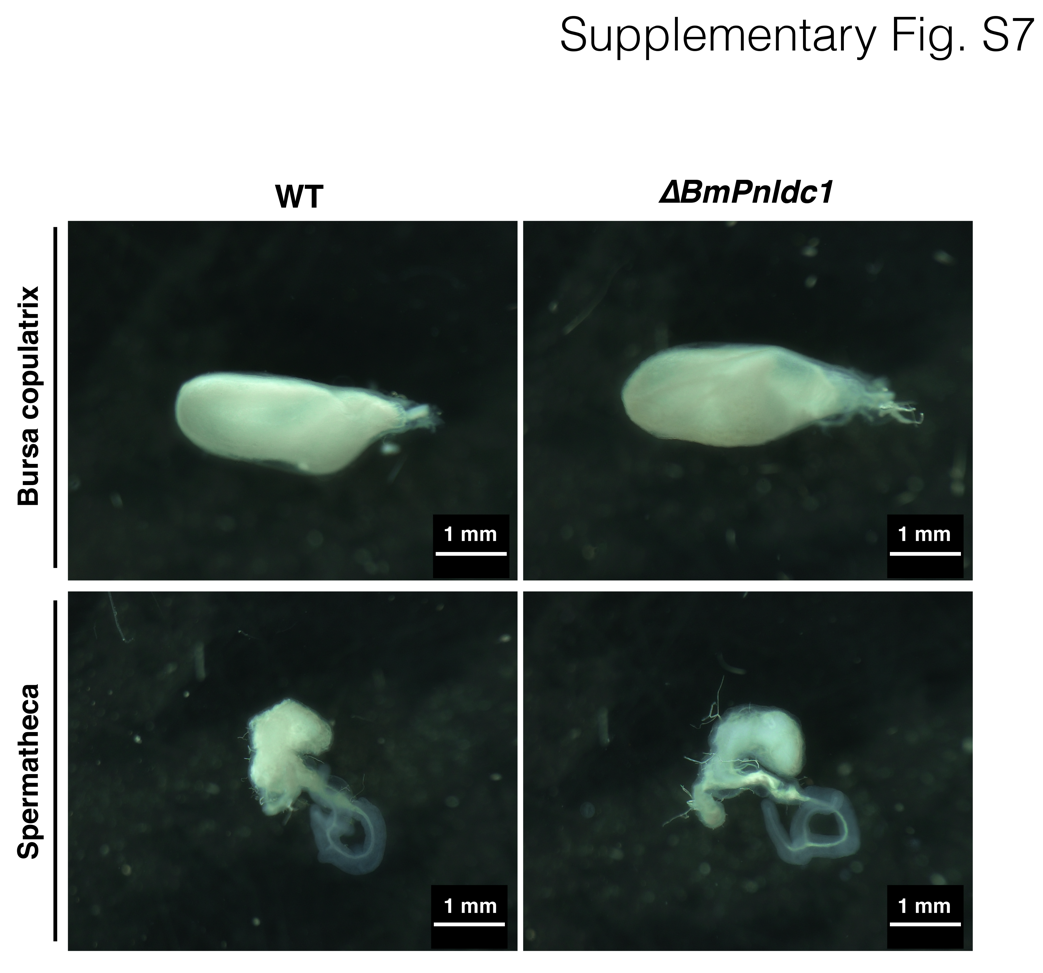


**Supplementary Fig. 7 Bursa copulatrix and spermatheca of females mated with WT males and *∆BmPnldc1* males (related to Fig. 6).** Scale bars, 1 mm.


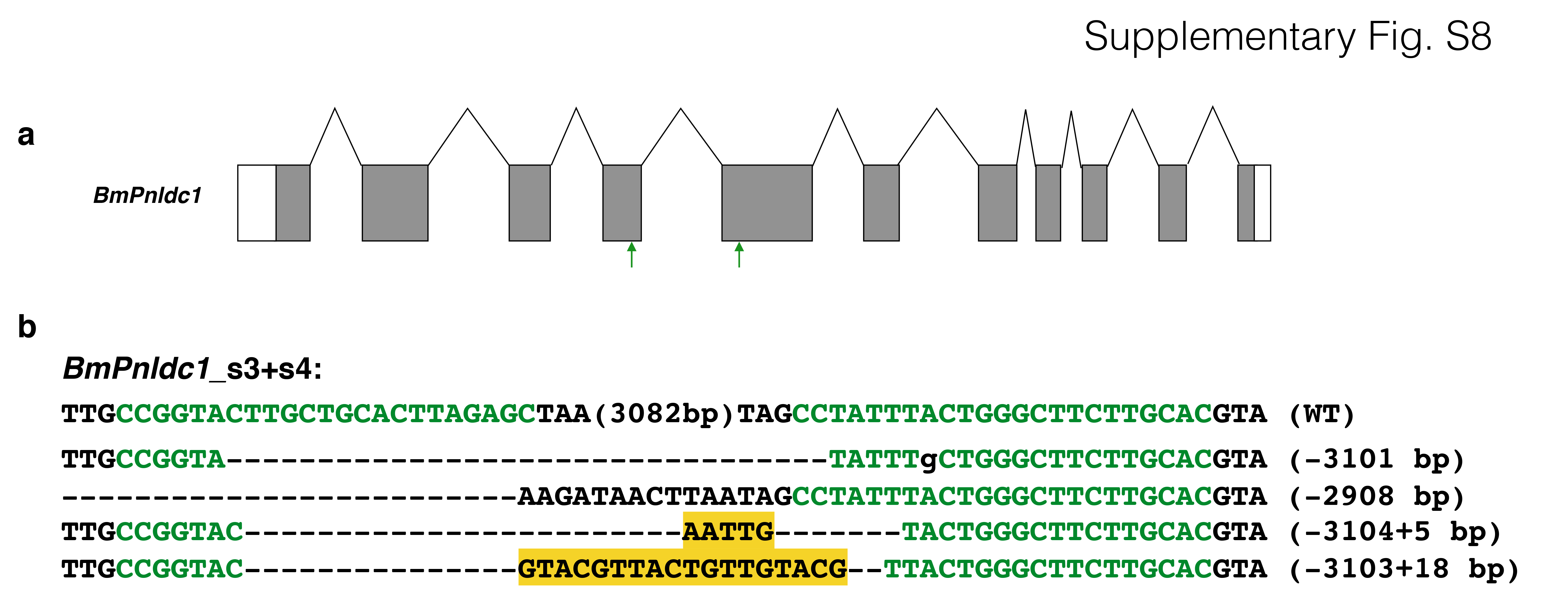


**Supplementary Fig. 8 Generation of** ***BmPnldc1*-sg34 × nos-Cas9 transgenic lines using CRISPR/Cas9 (related to Fig. 6).** **a** Schematic of *BmPnldc1* gene structure and sgRNA targets. Green arrows indicate the target sites of sgRNA3 and sgRNA4. **b** Genomic mutations of *BmPnldc1* gene. Mutations are shown with target sequences denoted in green. Deletions, dashed lines; insertions, yellow shadow; substitutions, lowercase letters. Indel events are shown to the right.


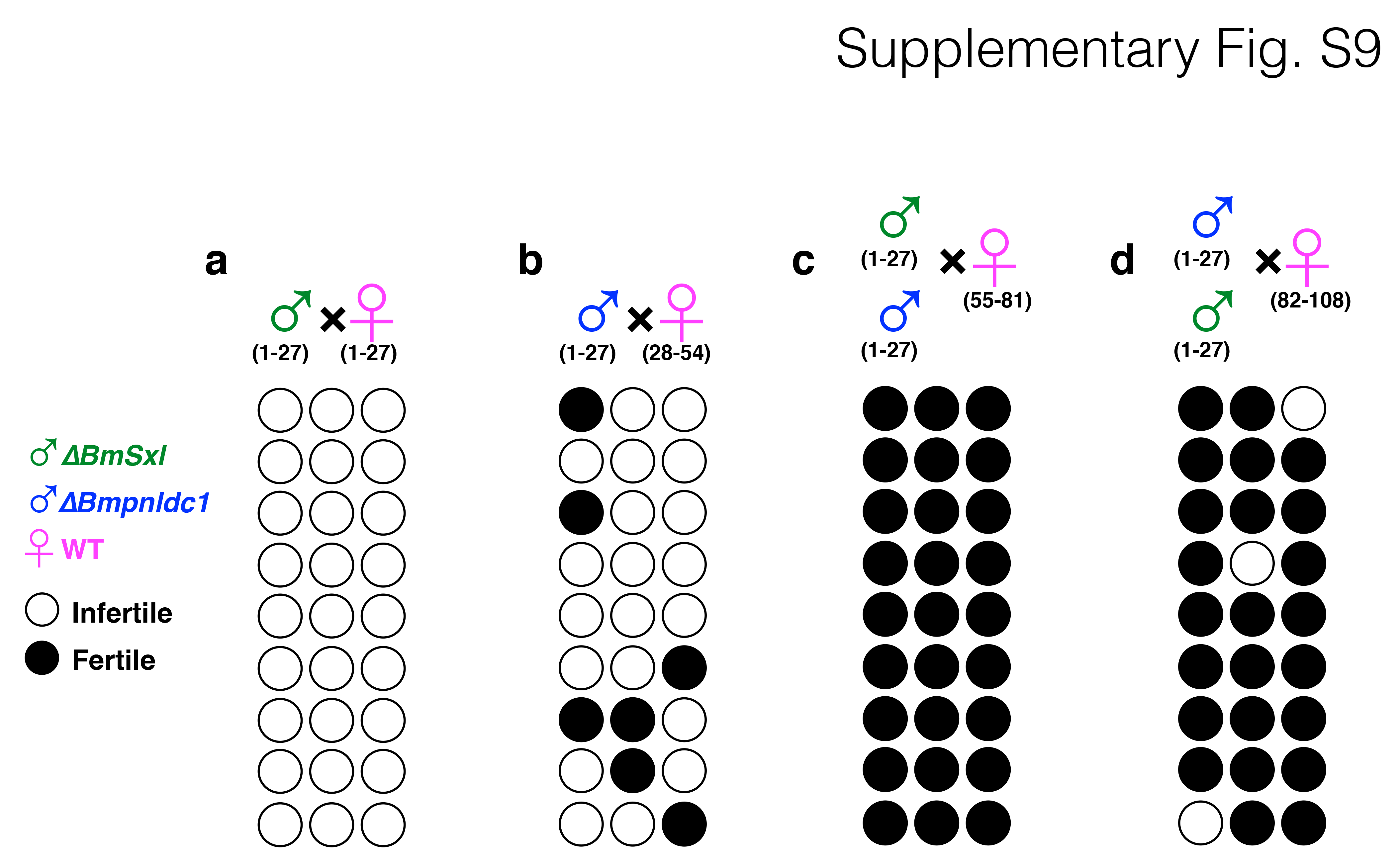


**Supplementary Fig. 9** **Diagrams illustrating fertility rescue assays (related to Fig. 7). a** Single copulation test conducted by mating of 1-27 *∆BmSxl* males with 1-27 WT virgin females for 3 h. The 27 broods were infertile. **b** Single copulation test conducted by mating of 1-27 *∆Bmpnldc1* males with 28-54 WT virgin females for 3 h. Seven out of the 27 broods were fertile. **c** Double copulation test conducted by mating of 55-81 WT virgin females with 1-27 *∆BmSxl* males in the first 3 h and with 1-27 *∆Bmpnldc1* males in the second 3 h. All 27 broods were fertile. **d** Double copulation test conducted by mating of 81-108 WT virgin females with 1-27 *∆Bmpnldc1* males in the first 3 h and with 1-27 *∆BmSxl* males in the second 3 h. Twenty-four of the 27 broods were fertile. Symbols on the left: green ♂, *∆BmSxl* males; blue ♂,*∆BmPnldc1* males; magenta ♀, WT females; filled circles, fertile broods; unfilled circles, infertile broods. Numbers in parentheses indicate the sequence numbers of the tested animals.

**Supplementary Table S1.** List of primers and gRNA target sequences used in this study.

| **Experiment** | **Primer/sgRNA targeting sequence name** | **sequence (5' to 3')** |
| --- | --- | --- |
| **Plasmid construction** | **KpnI-U6-F** | CTCACTATAGGGCGAATTGGAGGTTATGTAGTACACATTGTTGTA |
|  | **Overlap-gBone-R** | CCGCGGAGTCAATGGCTAGCAAAAAAAGCACCGACTCGGTG |
|  | **Overlap-U6-F** | GCTAGCCATTGACTCCGCGGAGGTTATGTAGTACACATTGTTGTA |
|  | **gBone-HindIII-R** | TTTTCTTGTTATAGATATCAAAAAAAAGCACCGACTCGGTG |
|  | **BmSxl-sg1-F** | CCGGCGGCCGAGAGTCCCCCACTTGTAGAGCACGATATTTTGTAT |
|  | **BmSxl-sg1-R** | GGGGGACTCTCGGCCGCCGGGTTTTAGAGCTAGAAATAGCAAGTT |
|  | **BmSxl-sg2-F** | CCTTCTGCTTGCCATGTTCCACTTGTAGAGCACGATATTTTGTAT |
|  | **BmSxl-sg2-R** | GGAACATGGCAAGCAGAAGGGTTTTAGAGCTAGAAATAGCAAGTT |
|  | **BmSxl-sg3-R** | TCTCTGTTATATTCTGAGGCACTTGTAGAGCACGATATTTTGTAT |
|  | **BmSxl-sg3-F** | GCCTCAGAATATAACAGAGAGTTTTAGAGCTAGAAATAGCAAGTT |
|  | **BmSxl-sg4-R** | GAGAGGAGTCGCCTTCGTTCACTTGTAGAGCACGATATTTTGTAT |
|  | **BmSxl-sg4-F** | GAACGAAGGCGACTCCTCTCGTTTTAGAGCTAGAAATAGCAAGTT |
|  | **BmPnldc1-sg1-F** | GAAATGTATATCCAGTTGCAGTTTTAGAGCTAGAAATAGCAAGTT |
|  | **BmPnldc1-sg1-R** | TGCAACTGGATATACATTTCACTTGTAGAGCACGATATTTTGTAT |
|  | **BmPnldc1-sg2-F** | GCTTTATACGGCTCGGTCACGTTTTAGAGCTAGAAATAGCAAGTT |
|  | **BmPnldc1-sg2-R** | GTGACCGAGCCGTATAAAGCACTTGTAGAGCACGATATTTTGTAT |
|  | **pcDNA3-KpnI-EGFP-F** | CTATAGGGAGACCCAAGCTTGGTACCATGGTGAGCAAGGGCGAGGA |
|  | **EGFP-linker-R** | AGAGCCGCCGCCGCCCTTGTACAGCTCGTCCATGC |
|  | **EGFP-linker-LY-F** | ACAAGGGCGGCGGCGGCTCTATGTGTGAACGTTATCCGGG |
|  | **EGFP-linker-SY-F** | ACAAGGGCGGCGGCGGCTCTATGTCTGAAGGAGGGGGACT |
|  | **AY-pcDNA3-XbaI-R** | GACACTATAGAATAGGGCCCTCTAGACTACCAATATGGCTGCGGGC |
|  | **BY-pcDNA3-XbaI-R** | CGAATAACAATGTGGCTTAAtctagaGGGCCCTATTCTATAGTGTC |
| **CRISPR sgRNA targeting sequences** | **BmSxl-sgRNA1** | GGGGGACTCTCGGCCGCCGGCGG |
|  | **BmSxl-sgRNA2** | GGAACATGGCAAGCAGAAGGCGG |
|  | **BmSxl-sgRNA3** | GCCTCAGAATATAACAGAGAAGG |
|  | **BmSxl-sgRNA4** | CCCGAGAGGAGTCGCCTTCGTTC |
|  | **BmPnldc1-sgRNA1** | CCTTGCAACTGGATATACATTTC |
|  | **BmPnldc1-sgRNA2** | GCTTTATACGGCTCGGTCACTGG |
|  | **BmPnldc1-sgRNA3** | CCGGTACTTGCTGCACTTAGAGC |
|  | **BmPnldc1-sgRNA4** | CCTATTTACTGGGCTTCTTGCAC |
| **Primers for qRT-PCR** | **C-F** | CAGCGTTAAAGTAGCAGAGG |
|  | **C-R** | GCAGTTCCACGAATAATCACC |
|  | **S-F** | AAAGACCAAATCCAACAGCA |
|  | **S-R** | TGTAGCCAGTCTTAAAGTCCT |
|  | **L-F** | AGCTGGGACAGGAGTTTCAA |
|  | **L-R** | TGGTAACGAACATCGCGTAA |
|  | **A-F** | TTCGTTTGGATTGTGGATCA |
|  | **A-R** | CGTGCAACAAAGTTGCGTAT |
|  | **B-F** | GCAGAAGGCGGCTTATTATG |
|  | **B-R** | TGTTATTCGCTCGACCTCCT |
|  | **Bmrp49-qRT-F** | TCAATCGGATCGCTATGACA |
|  | **Bmrp49-qRT-R** | ATGACGGGTCTTCTTGTTGG |
|  | **pyruvate kinase-F** | GCCAGTGCCTGTAAAGAAGC |
|  | **pyruvate kinase-R** | ATACAATTTGCGCCGATTTC |
|  | **glutamine synthetase 1-F** | AATTTGAAACGGCATCGTTC |
|  | **glutamine synthetase 1-R** | GGAGTAGCCCACAAACCAAA |
|  | **pyruvate kinase-like-F** | CCCCTTTGAACATCGAAGAA |
|  | **pyruvate kinase-like-R** | GCCAATCTGAAGTCCCATGT |
|  | **L-threonine ammonia-lyase-F** | GGGAGGATATCGTCGTCAGA |
|  | **L-threonine ammonia-lyase-R** | GGGTTCGACTGTTTCATCGT |
|  | **aminoacylase-1-F** | AGGGTTGATTGCTGGCATAG |
|  | **aminoacylase-1-R** | CGCTGTGTAAAAAGCGTTGA |
|  | **L-threonine ammonia-lyase-like-F** | CACCCCATCCCATACTCAAC |
|  | **L-threonine ammonia-lyase-like-R** | TCGTTTTTATCGCTGCACTG |
|  | **ornithine carbamoyltransferase-like-F** | ATTCCGCTTGTTGAAGCAGT |
|  | **ornithine carbamoyltransferase-like-R** | GGCTTGTCCATTTCCAAAGA |
|  | **L-threonine dehydratase catabolic TdcB-F** | ATTCCGCTTGTTGAAGCAGT |
|  | **L-threonine dehydratase catabolic TdcB-R** | GGCTTGTCCATTTCCAAAGA |
|  | **pyruvate carboxylase-F** | ATCTCTCACGCTCACGACCT |
|  | **pyruvate carboxylase-R** | CTTCTGCGCTCTGTTCTGTG |
|  | **phosphoribosyl pyrophosphate synthase-F** | CCAGGTCGCGTACTATGGAT |
|  | **phosphoribosyl pyrophosphate synthase-R** | TGTAGGCTCCGCACTCTTTT |
|  | **fructose-bisphosphate aldolase-F** | GGGCATTGGAAAGCTACTGA |
|  | **fructose-bisphosphate aldolase-R** | AACGCCGTGACATCCTTATC |
|  | **NADP-dependent malic enzyme-F** | TGTTGTGGGTGGTGCTTTTA |
|  | **NADP-dependent malic enzyme-R** | GCGTTGTTACCCTGTCCAGT |
|  | **malate dehydrogenase-F** | CATGCTCTCCTCTGTCACGA |
|  | **malate dehydrogenase-R** | AATGCTCAAAAGCCAAATCG |
|  | **glutamate dehydrogenase-F** | AGATGCAGGGCTTCAAGTGT |
|  | **glutamate dehydrogenase-R** | AGGATGGTGGTCCTGAAGTG |
|  | **phosphoglycerate dehydrogenase-F** | CACGTTGATGTTGGTTCTGG |
|  | **phosphoglycerate dehydrogenase-R** | TGATCAGCAGACACGAAAGG |
|  | **tyrosine aminotransferase-F** | TTCCTCGACCAGGGTTTATG |
|  | **tyrosine aminotransferase-R** | TGTACACGGATCCACAAGGA |
|  | **BmPnldc1-qRT-PCR-F** | GTTCAAATACAGCTTGTTCGACAC |
|  | **BmPnldc1-qRT-PCR-R** | CAATGCCTCATCACTCTTGCT |
| **Mutagenesis detection** | **BmSxl-ko-F1** | GGGCCCACTTTGATCTCGTA |
|  | **BmSxl-ko-R1** | CTGAGGTATCACCGGAGCTGAATC |
|  | **BmSxl-ko-R2** | TATTGCCTCTTGCGCTTCTT |
|  | **BmPnldc1-ko-F** | ATGGATATCACCAAAGAAAA |
|  | **BmPnldc1-ko-R1** | TACCGTCGCTCATGGACAGC |
|  | **BmPnldc1-ko-R2** | GACAGGTCTGTTCCGAGGTG |
